# Development of an isavuconazole physiologically-based pharmacokinetic model for adult and pediatric populations

**DOI:** 10.1007/s10928-026-10061-8

**Published:** 2026-09-22

**Authors:** Mary P Choules, Amit Desai, Laura Lynn Kovanda, Yukio Otsuka, Roger J Brüggemann, Peter L Bonate

**Affiliations:** 1 Astellas Pharma Global, Inc, 2375 Waterview Dr, Northbrook, IL 60062 USA; 2 Astellas Pharma, Inc, Tokyo, Japan; 3 Department of Pharmacy, Pharmacology and Toxicology, and Radboudumc- CWZ Center of Expertise in Mycology, Radboud University Medical Center, Nijmegen, The Netherlands

**Keywords:** Physiological-based pharmacokinetics (PBPK), Drug-drug interaction (DDI), Simcyp, Isavuconazole, Antifungal, Pediatric DDI

## Abstract

Isavuconazonium sulfate is the water-soluble prodrug of the active triazole antifungal agent isavuconazole. Isavuconazonium sulfate is approved for the treatment of invasive aspergillosis and invasive mucormycosis in pediatric and adult populations. In the present analysis, an isavuconazole physiologically-based pharmacokinetic (PBPK) model was built and verified in adult and pediatric populations using the Simcyp^®^ simulator. The verified pediatric model was then used to predict the magnitude of DDI risk in pediatric populations vs. adult populations. The simulations conducted were designed to match the observed clinical trials conducted in adults but with pediatric equivalent doses based on clinical guidelines. Based on the results of the analysis, isavuconazole DDI risk was similar between pediatric and adult populations. When the isavuconazole perpetrator DDI simulations were compared to adults, there was a trend of larger geometric mean (GM) AUC_inf_ and C_max_ ratios in patients 1 to < 3 years old receiving the 15 mg/kg dose. The GM AUC_inf_ ratio in this study population ranged from 7 to 36% larger than predicted in the adult population. Based on the results of this analysis and literature reports, it is advisable to show greater caution when considering potential DDI in patients < 3-year-old, especially when receiving larger isavuconazonium sulfate doses. In addition, caution should be used in patients < 3 years-old when considering coadministration of isavuconazole with narrow therapeutic index medications such as digoxin and warfarin or medications with large variability in oral bioavailability such as midazolam.

**Supplementary Information:**

The online version contains supplementary material available at https://doi.org/10.1007/s10928-026-10061-8.

## Introduction

Isavuconazonium sulfate is the water-soluble prodrug of the active triazole isavuconazole, which is an antifungal agent that inhibits sterol 14α-demethylase, a microsomal P450 enzyme essential for ergosterol biosynthesis in fungi [1, 2]. The oral (po) and intravenous (iv) formulations of isavuconazonium sulfate were approved by FDA and EMA in 2015 for the treatment of invasive aspergillosis (IA) and invasive mucormycosis (IM) in adults at a dose of 372 mg every 8 h (Q8H) for 6 doses followed by once daily dosing. Isavuconazonium sulfate was approved by FDA in December 2023 at a dose of 10 mg/kg (15 mg/kg in ages 1 to 3 Q8H) for the first 6 doses (2 days), then daily as iv administration for use in children 1 year of age or older. The oral formulation was approved for children > 6 years old and ≥ 16 kg. In August 2024, isavuconazole was approved for use in pediatric populations (ages 1 to < 18 years) by EMA. For patients that weigh > 37 kg the same dosing regimen as adults is used. For patients that weigh < 37 kg, 10 mg/kg Q8H for the first 6 doses followed by daily dosing. The maximum dose for all populations is 372 mg isavuconazonium sulfate as either oral capsule or iv administration. The established steady-state AUC_24h_ target concentration is 60 mg·h/L to 233 mg·h/L [3] with goal trough levels estimated between 2 and 5 mg/L [4].

Upon administration, isavuconazonium sulfate is rapidly converted by plasma esterases, primarily butyrylcholinesterase (BChE), to isavuconazole. The dose conversion of the prodrug isavuconazonium sulfate to active moiety isavuconazole is 372 mg isavuconazonium sulfate to 200 mg isavuconazole [1, 2]. Isavuconazonium sulfate is generally classified as a Biopharmaceutical Classification System (BCS) Class III drug substance, with high solubility and low permeability. However, due to the rapid conversion to isavuconazole, which exhibits rapid absorption, it acts as a BCS Class I drug in vivo. Isavuconazole is primarily metabolized by CYP3A4/5 and secondarily by UGT with a large fraction (46.1%) eliminated in the feces via biliary elimination. Isavuconazole was not found to be a substrate of P-glycoprotein (P-gp), breast cancer resistance protein (BCRP), organic anion transporting polypeptide (OATP)1B1 or OATP1B3 [1]. Clinically, isavuconazole was found to be a moderate inhibitor of CYP3A4, an inducer of CYP2B6, and has inhibitory effects on P-gp and OCT2 transporters [1, 2].

Triazole antifungal compounds, such as isavuconazole, are the most frequently prescribed antifungals; however, they are highly prone to drug-drug interactions (DDIs) [5]. Physiologically-based pharmacokinetic (PBPK) modeling has been historically implemented to predict the magnitude of DDI for medication combinations where clinical DDI studies may be difficult to conduct or to fill in gaps in knowledge based on previously conducted studies [6, 7].Several PBPK models for the triazole compounds have been developed over the years to predict potential DDIs and to optimize dosing in pediatric populations [8–11]. Although several clinical DDI studies were conducted in adult populations during isavuconazole development, no DDI studies were conducted in pediatric populations. With the approval of isavuconazole in pediatric populations, an understanding of isavuconazole DDI risk in pediatric populations is essential for clinical use due to the metabolic differences between adults and children [12]. In the present analysis, we report on the construction and verification of an isavuconazole PBPK model in adult and pediatric populations. The verified pediatric isavuconazole model was then used to predict the potential DDI when isavuconazole is coadministered with the same inhibitors and substrates utilized in the adult clinical studies.

## Methods

**Fig. 1:**
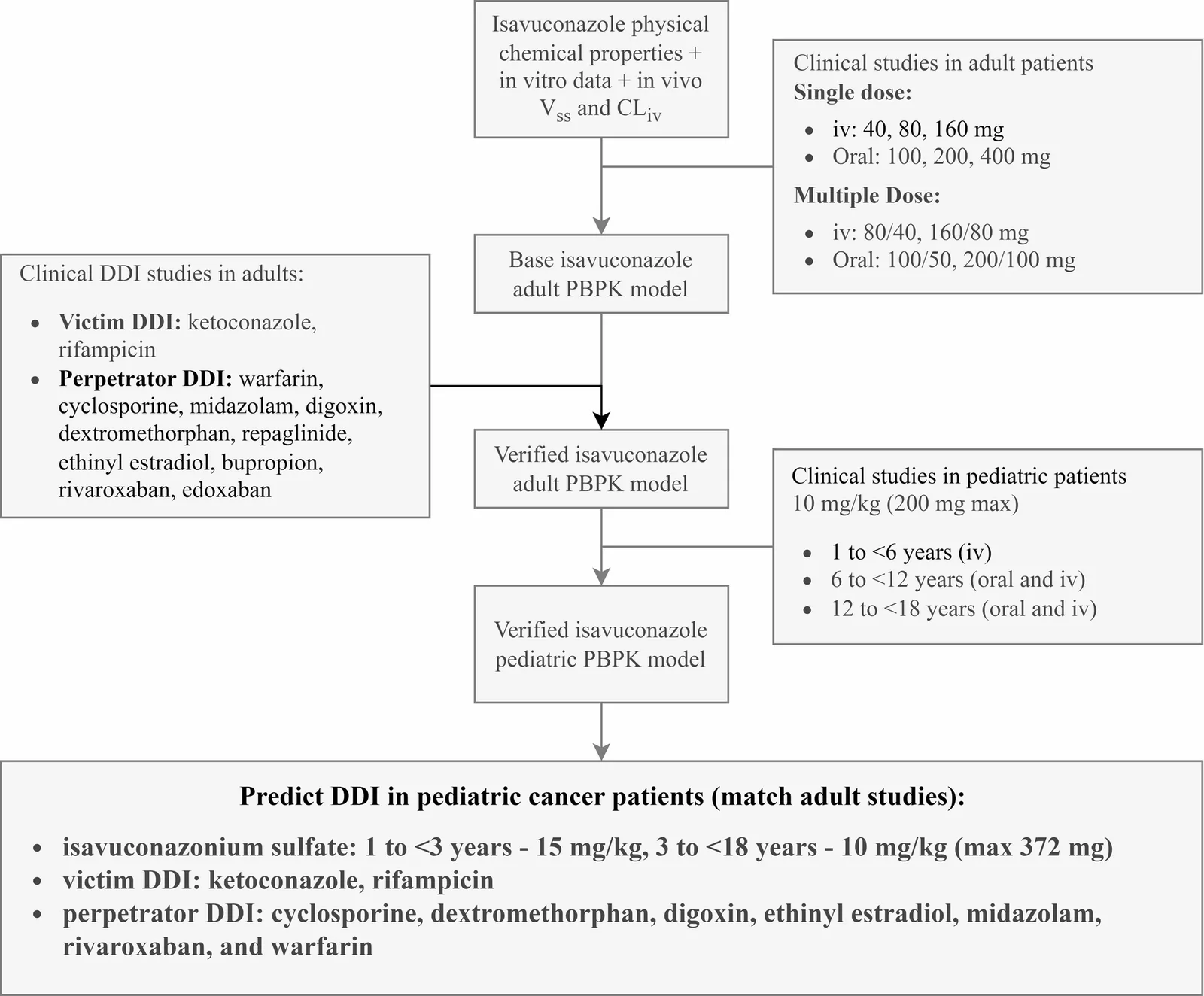
Overall workflow for the building, verification, and implementation of isavuconazole PBPK model

A summary of the overall modeling workflow can be found in Fig. 1. The PBPK model was designed so that only the active moiety, isavuconazole, was administered instead of the inactive prodrug, isavuconazonium sulfate. This approach was considered reasonable due to the rapid and complete absorption of isavuconazonium sulfate followed by its rapid and complete conversion to isavuconazole. As shown in the figure, the isavuconazole PBPK model was built using a hybrid bottom-up approach with base model parameters obtained from in vitro experiments and in vivo PK parameters (V_ss_ and CL_iv_). Then a top-down approach utilizing observed clinical data was implemented for model optimization. The model was first verified in adult populations then translated and verified for pediatric populations. Model building and verification were carried out using observed clinical PK data from both adult and pediatric patients. The resulting verified model was then used to predict various DDIs in pediatric patients. More details on the model building and verification can be found in the “Isavuconazole PBPK Model” section.

All simulations were carried out in the SimcypSimulator (Certara, Sheffield UK, version 24). The Simcyp simulator-provided PBPK models for cyclosporine, bupropion, dextromethorphan, digoxin, ethinyl estradiol, ketoconazole, midazolam, rifampicin, repaglinide, verapamil, and S-warfarin were also used without any modifications. Literature referenced plasma concentration data were extracted using GetData Graph Digitizer (http://getdata-graph-digitizer.com, version 2.26.0.20). All virtual trial designs mimicked the observed clinical trials. Details about the clinical data utilized can be found in the supplemental information Tables S1 and S2. All reported simulated pharmacokinetic (PK) and statistical parameters were calculated by the simulator. Model performance was determined by calculating the geometric mean fold error (GMFE) (Eq. 1) and through the Guest criteria (Eq. 2) assuming 20% variability in pharmacokinetic parameters [13]. Where *δ* is a parameter that accounts for variability (1.25 for 20% variability), *R*_*obs*_ is defined as AUC_+inhibitor_/AUC_control_ ≥ 1 for inhibition DDIs. For induction DDIs, *R*_*obs*_ was calculated using the reciprocal (AUC_control_/AUC_+inducer_). The upper limit was calculated as *R*_obs_* Limit and the lower limit was calculated as *R*_*obs*_*/Limit* where *R*_*obs*_ is AUC_+inhibitor/inducer_/AUC_control_.

1 $$\boldsymbol{G}\boldsymbol{M}\boldsymbol{F}\boldsymbol{E}=\mathbf{exp}\left(\frac{1}{\boldsymbol{n}}\sum\:_{\boldsymbol{i}=1}^{\boldsymbol{n}}\left|\boldsymbol{l}\boldsymbol{n}\left(\frac{{\boldsymbol{P}\boldsymbol{r}\boldsymbol{e}\boldsymbol{d}\boldsymbol{i}\boldsymbol{c}\boldsymbol{t}\boldsymbol{e}\boldsymbol{d}}_{\boldsymbol{i}}}{{\boldsymbol{O}\boldsymbol{b}\boldsymbol{s}\boldsymbol{e}\boldsymbol{r}\boldsymbol{v}\boldsymbol{e}\boldsymbol{d}}_{\boldsymbol{i}}}\right)\right|\right)$$

2 $$\:\boldsymbol{L}\boldsymbol{i}\boldsymbol{m}\boldsymbol{i}\boldsymbol{t}=\:\frac{\boldsymbol{\delta\:}+2\left({\boldsymbol{R}}_{\boldsymbol{o}\boldsymbol{b}\boldsymbol{s}}-1\right)}{{\boldsymbol{R}}_{\boldsymbol{o}\boldsymbol{b}\boldsymbol{s}}}$$

### Isavuconazole PBPK model

A summary of the model parameters used in the final isavuconazole PBPK model can be found in Table 1. The isavuconazole model was built using 1st order absorption model and the full PBPK model for distribution with the tissue-to-plasma partition coefficient (Kp) scalar set to 1 (V_ss_ = 4.48 L/kg) based on observed data and was consistent with the reported volume of distribution (135 to 433 L). Intrinsic clearance (CL_int_) was calculated using the Simcyp retrograde calculator from a CL_iv_ of 2.36 L/h, which was the mean estimate from a population pharmacokinetic (PopPK) analysis for isavuconazole clearance across all Phase 1 to Phase 3 clinical trials [22]. Percent biliary clearance of isavuconazole was reported in a mass balance study where 46.1% of the administered dose (200 mg equivalent dose) radioactivity was recovered in the feces [23]. An internal metabolite analysis estimated that 7.84% of the excreted radioactivity in the feces was unchanged isavuconazole. The same mass balance study recovered 45.5% of total radioactivity in the urine and a renal impairment study reported the total renal clearance of isavuconazole to be 0.0125 L/h [24]. The average total recovery from this mass balance study was 91.6% (range 86.3–96.7%). The mass balance of 200 mg oral isavuconazole was assessed using the PBPK model with the same virtual trial design as the clinical trial. After a 600-hour simulation, the total recovery was 97.2% with 88% of the administered dose metabolized, then 8.6% and 0.527% excreted unchanged in the feces and urine, respectively.

**Table 1:** Input parameters for the isavuconazole PBPK model

Parameter		Value	Assumption(s) and references
Compound type		Small molecule	
Molecular weight		437.47	[1]
Log P		4.1	[1]
Charge		Neutral	[1]
B/P ratio		0.6	Internal data
f_u_		0.00737	Internal data
Plasma binding protein		HSA	[1]
Absorption			
Absorption Model		First-Order	
Lag time (h)		1.5	Optimized to adult oral administration
P_eff, man_ (10^− 4^ cm/s)		3.33	Predicted by Simcyp
Caco-2 (10^− 6^ cm/s) Apical pH : Basolateral pH		13.5 7.4 7.4	Internal in vitro Caco-2 data (reference: propranolol – 18.7·10^− 6^ cm/s)
Distribution			
Distribution model		Full PBPK	
Prediction method of V_ss_		Method 2	Rodgers
V_ss_ (L/kg)		4.48	Predicted by Simcyp
K_p_ scalar		1	Default
Elimination			
CL_R_ (L/h)		0.0125	[24]
CYP3A4 CL_int_ (µL/min/pmol)		0.646	retrograde analysis: CL_iv_ 2.36 L/h, f_m, CYP3A4_ 0.87
CYP3A5 CL_int_ (µL/min/pmol)		0.0333	retrograde analysis: CL_iv_ 2.36 L/h, f_m, CYP3A5_ 0.04
Additional HLM CL_int_ (µL/min/mg protein)		0.567	retrograde analysis: CL_iv_ 2.36 L/h
CL_int_ (Bile) (µL/min/10^6^)		2.57	retrograde analysis: CL_iv_ 2.36 L/h, f_CL, Bile_ 7.84%
Interaction			
CYP1A2	Ind_max_	6.63	Calculated with internal in vitro induction data
	Ind_C50_ (µmol/L)	2.83	
CYP2B6	Ind_max_	12.2	Optimized based on bupropion DDI [47]
	Ind_C50_ (µmol/L)	0.250	
CYP2C8	K_i_ (µmol/L)	2.86	Internal in vitro inhibition data
	Ind_max_	5.40	Calculated with internal in vitro induction data
	Ind_C50_ (µmol/L)	0.540	
CYP2C9	K_i_ (µmol/L)	0.280	Optimized based on warfarin DDI [48]
	Ind_max_	2.47	Calculated with internal in vitro induction data
	Ind_C50_ (µmol/L)	0.880	
CYP2C19	K_i_ (µmol/L)	5.40	Internal in vitro inhibition data
CYP2D6	K_i_ (µmol/L)	0.270	Optimized based on dextromethorphan DDI [47]
CYP3A4	K_i_ (µmol/L)	0.180	Optimized based on cyclosporine DDI [49]
	Ind_max_	9.47	Calculated with internal in vitro induction data
	Ind_C50_ (µmol/L)	5.60	
CYP3A5	K_i_ (µmol/L)	0.180	Assumed same as CYP3A4
	Ind_max_	9.47	
	Ind_C50_ (µmol/L)	5.60	
P-gp	K_i_ (µmol/L)	0.0100	Optimized based on digoxin DDI [50]

*LogP* octanol: buffer partition coefficient, *B/P ratio* blood-to-plasma partition ratio, *f*_*u*_ fraction unbound, *HSA* Human Serum Albumin, *P*_*eff, man*_ Human jejunum effective permeability, *V*_*ss*_ volume of distribution at stead-state, *K*_*p*_ *scalar* scalar for all tissue: plasma partition coefficients, *CL*_R_ renal clearance, *CL*_*int*_ intrinsic clearance, *f*_*m, CYPX,*_ fraction metabolized by CYP enzyme X, *HLM* human liver microsomes, *K*_*i*_ concentration of inhibitor that supports half maximal inhibition, *Ind*_*max*_ maximal fold induction over vehicle, *Ind*_*C50*_ test compound concentration that supports half maximal induction

Isavuconazole was found to be primarily metabolized by CYP3A4 and to a lesser extent CYP3A5. Isavuconazole was not found to be the substrate of P-gp, breast cancer resistance protein (BCRP), organic anion-transporting polypeptide (OATP)1B1 or OATP1B3 [1]. An in vitro assay found that in an incubation mixture with CYP3A4 vs. CYP3A5 expressing microsomes, 33.8% vs. 68.4% isavuconazole was remaining after 30 min, respectively. The Michaelis-Menten kinetic constants and the fraction metabolized (f_m_) through CYP3A4 or CYP3A5 for isavuconazole were not specifically determined with in vitro analysis. Therefore, f_m, CYP3A4_ and f_m, CYP3A5_ of isavuconazole were estimated and verified by capturing clinical DDI data between isavuconazole and the strong CYP3A inhibitor ketoconazole and inducer rifampicin. Both ketoconazole and rifampicin inhibit or induce both CYP3A4 and CYP3A5.

Isavuconazole was found to have inhibitory activities in vitro against CYP3A4/5 with the K_i_ of 0.622 to 1.93 µmol/L, CYP2C8 with K_i_ of 2.86 µmol/L, CYP2C9 with K_i_ of 4.78 µmol/L, CYP2C19 with K_i_ of 5.40 µmol/L and CYP2D6 with K_i_ of 4.82 µmol/L (internal data). Isavuconazole was also found to induce the activity of CYP1A2, CYP2B6, CYP2C8, and CYP3A4/5 by < 10%, 84.3%, 37.4% and 42.2%, respectively. Finally, isavuconazole was found to inhibit the transporters P-gp (IC_50_ 25.7 µmol/L), organic cation transporter (OCT)1 (K_i_ 1.74 µmol/L), OCT2 (K_i_ 0.590 µmol/L), OATP1B1 (IC_50_ 11.2 µmol/L) and multidrug and toxin extrusion protein (MATE)1 (IC_50_ 6.31 µmol/L) (internal data). Virtual trial designs were created to optimize and verify the inhibition or induction potential of isavuconazole against the various CYP enzymes. DDI predictions were conducted for bupropion, cyclosporine, dextromethorphan, digoxin, edoxaban, ethinyl estradiol, midazolam, repaglinide, rivaroxaban and warfarin. To aid in verification on K_i, P-gp_, an edoxaban PBPK model was constructed. Details on its construction and verification can be found in the edoxaban section of the supplemental information. In short, the model was constructed based on the published PBPK model by Xu R, et al. and verified using published PK and DDI data [25]. The clinical data utilized in the model building and verification can be found in Table S9 and the final model parameters can be found listed in Table S10.

When the model was translated to pediatrics, all drug model parameters remained the same as the adult model. The CL_int_ for CYP3A4 and CYP3A5 that was calculated for the adult model remained the same for the pediatric model. Altering CL_int_ was not required since simulated total clearance is dependent on the population CYP abundance for each simulated patient. For the pediatric simulations, the designated ontogeny (discussed more in the Population models section) drives the virtual patient CYP abundance. Of note, due to the protocol detailing the clinical trial study design, participants were allowed to receive their first maintenance dose (starting on day 3 of dosing) anywhere from 12 to 24 h following their last loading dose. Therefore, participants were split into two categories based on if they receive their first maintenance dose 12–24 h following the last loading dose. PK parameters were summarized based on a combination of these categories while observed versus simulated plasma concentration-time curves remain separated.

### Population models

The Simcyp simulator provided PBPK population model for healthy volunteer population and cancer population were used without any modifications for most simulations. For the simulations in pediatric populations, the pediatric hematologic cancer population was used with minor modifications. The pediatric ontogeny followed the equations detailed in Eq. 3 and Eq. 4 where, *Adult*_*Max*_ = fraction of maximum expression reached compared to adults, *F*_*Birth*_ = fraction at birth, *Age*_*50*_ *=* age to half maximum, *n* = exponent, *Age* = post-natal age in years, and *Age-cap* = age at which the *Adult*_*Max*_ is reached [11, 14]. The ontogeny for CYP3A4 was changed from the default profile 1 in Simcyp (reported by Salem et al., [15, 16]) to profile 2 (reported by Upreti and Wahlstrom [12]). The only value that was altered from the default profile 2 was the *Age-cap*^*2*^, the value was increased from 12.5 to 25, which is the same as the Japanese pediatric population in Simcyp. Age-cap^2^ was included to stop the function from going below one. Increasing age-cap^2^ stops the function from going below 1 and prolongs the CYP3A4 elevations which is consistent from literature reports showing increased clearance of midazolam even past 16 years old [12].

3 $$\boldsymbol{F}\boldsymbol{r}\boldsymbol{a}\boldsymbol{c}\boldsymbol{t}\boldsymbol{i}\boldsymbol{o}\boldsymbol{n}\:\boldsymbol{o}\boldsymbol{f}\:\boldsymbol{a}\boldsymbol{d}\boldsymbol{u}\boldsymbol{l}\boldsymbol{t}=\:\left(\frac{{\boldsymbol{A}\boldsymbol{d}\boldsymbol{u}\boldsymbol{l}\boldsymbol{t}}_{\boldsymbol{M}\boldsymbol{a}\boldsymbol{x}}-{\boldsymbol{F}}_{\boldsymbol{B}\boldsymbol{i}\boldsymbol{r}\boldsymbol{t}\boldsymbol{h}}}{{\boldsymbol{A}\boldsymbol{g}\boldsymbol{e}}_{50}^{\boldsymbol{n}}+\:{\boldsymbol{A}\boldsymbol{g}\boldsymbol{e}}^{\boldsymbol{n}}}\right)\times\:\:{\boldsymbol{A}\boldsymbol{g}\boldsymbol{e}}^{\boldsymbol{n}}+\:{\boldsymbol{F}}_{\boldsymbol{B}\boldsymbol{i}\boldsymbol{r}\boldsymbol{t}\boldsymbol{h}}$$

4 $$\boldsymbol{F}\boldsymbol{r}\boldsymbol{a}\boldsymbol{c}\boldsymbol{t}\boldsymbol{i}\boldsymbol{o}\boldsymbol{n}={\boldsymbol{C}}_{0}+{\boldsymbol{C}}_{1}\bullet\:{\boldsymbol{e}\boldsymbol{x}\boldsymbol{p}}^{{\boldsymbol{C}}_{2}\left(\boldsymbol{A}\boldsymbol{g}\boldsymbol{e}-{\boldsymbol{A}\boldsymbol{g}\boldsymbol{e}-\boldsymbol{c}\boldsymbol{a}\boldsymbol{p}}^{1}\right)}$$

No ontogeny differences were included for P-gp transporter activity as literature reports that ABCB1 mRNA expression is completed relatively soon after birth. Expression was independent of age from 4 months to 20 years of age with P-gp expression levels not found to be significantly different [17–19]. Although expression immediately after birth to < 1 year was found to be lower than adults, this difference does not impact the present analysis. In addition, due to the complexity of CYP3A5 expression, no specific ontogeny was included for CYP3A5, instead the CYP3A4-CYP3A5 correlation factor built within Simcyp was implemented. Based on literature data, CYP3A5 expression has been found to be correlated with CYP3A4 expression and abundance [20, 21].

### Prediction of isavuconazole DDI risk in pediatric population

Simulations were conducted to assess the DDI risk in a pediatric cancer population with the inhibitor ketoconazole, the inducer rifampicin, and the substrates cyclosporine, dextromethorphan, digoxin, ethinyl estradiol, midazolam, rivaroxaban, and warfarin. For every simulation, virtual trial designs were set up with 10 trials with 10 subjects in each trial and 0.5 proportion of females. For every simulation, isavuconazole was administered by iv administration over 1 h with a dose of 8.06 mg/kg (15 mg/kg isavuconazonium sulfate) for ages 1 to < 3 years old and 5.38 mg/kg (10 mg/kg isavuconazonium sulfate) for ages 1 to < 18 years old with a maximum dose of 200 mg (372 mg isavuconazonium sulfate). Simulations were conducted for both 15 mg/kg and 10 mg/kg isavuconazonium sulfate doses in the 1 to < 3 years old population. Pediatric age ranges were split up into 1 to < 3 years, 3 to < 6 years, 6 to < 12 years, and 12 to < 18 years. The doses of inhibitor/inducer or substrate were selected based on the FDA pediatric prescribing information or literature reported for each drug except for rivaroxaban, since the study conducted in adults was microdose study, the microdose was also used in the pediatric predictions for comparison purposes. No simulations were conducted for ages 1 to < 3 years for dextromethorphan and ethinyl estradiol since clinical use is not expected/contraindicated. The simulation for ethinyl estradiol was conducted in subjects aged 12 to < 18 years old with the same dose as the adult clinical trial. The virtual trials were designed to match the clinical studies conducted in adults and were as follows:

- **Single dose ketoconazole**: 6.6 mg/kg oral ketoconazole [26] given on day 1 at the same time as isavuconazole.
- **Multiple dose ketoconazole**: 6.6 mg/kg oral ketoconazole [26] daily for 24 days with isavuconazole given simultaneously on day 4.
- **Multiple dose ketoconazole + multiple dose isavuconazole**: 6.6 mg/kg ketoconazole daily [26] for 24 days. Isavuconazole starting on day 9 given 2 h after the ketoconazole dose, Q8H for 2 days followed by daily administration to day 22.
- **Multiple dose rifampicin + multiple dose isavuconazole**: 10 mg/kg rifampicin [27] (dose max 600 mg) daily for 24 days. Isavuconazole starting on day 9 given 2 h after the ketoconazole dose, Q8H for 2 days followed by daily administration to day 22.
- **Cyclosporine**: Isavuconazole Q8H for 2 days followed by daily administration to day 8. 4 mg/kg oral cyclosporine [28, 29] given simultaneously with isavuconazole on day 5.
- **Dextromethorphan**: Isavuconazole Q8H for 2 days followed by daily administration to day 7. 11 mg (corrected for salt for 15 mg) oral dextromethorphan [30] given simultaneously with isavuconazole on day 5.
- **Digoxin**: Isavuconazole Q8H for 2 days followed by daily administration to day 12. 6 µg/kg (1 to < 3 years) or 5 µg/kg (3 to < 18 years) oral digoxin [31] given simultaneously with isavuconazole on day 5.
- **Ethinyl estradiol**: Isavuconazole Q8H for 2 days followed by daily administration to day 8. 35 µg oral ethinyl estradiol given simultaneously with isavuconazole on day 5.
- **Midazolam**: Isavuconazole Q8H for 2 days followed by daily administration to day 12. 1 mg/kg (20 mg dose maximum) oral midazolam [32] given simultaneously with isavuconazole on day 11.
- **Rivaroxaban**: Isavuconazole Q8H for 1 day. 25 µg oral rivaroxaban given 1 h after the first isavuconazole dose on day 1.
- **Warfarin**: Isavuconazole Q8H for 2 days followed by daily administration to day 13. 0.2 mg/kg oral warfarin (10 mg dose max) [33] given simultaneously with isavuconazole on day 5.

### Sensitivity analysis

Sensitivity analyses were undertaken to evaluate values 10-fold higher and 10-fold lower than the value used in the isavuconazole and for K_i, CYP3A4_ (0.018-1.8 µmol/L), K_i, CYP3A5_ (0.018-1.8 µmol/L, and K_i, P-gp, liver_ (0.001-0.1 µmol/L). The clinical DDI studies with midazolam and cyclosporine (study 2) were used for the K_i, CYP3A4/5_ sensitivity analyses. The clinical DDI studies with digoxin and edoxaban were used for the K_i, P-gp_ sensitivity analysis. The subject representative selected by the simulator was 23.5 years old and was a CYP3A5 poor metabolizer for K_i, CYP3A4_ and extensive metabolizer for K_i, CYP3A5_ assessment.

## Results

**Fig. 2:**
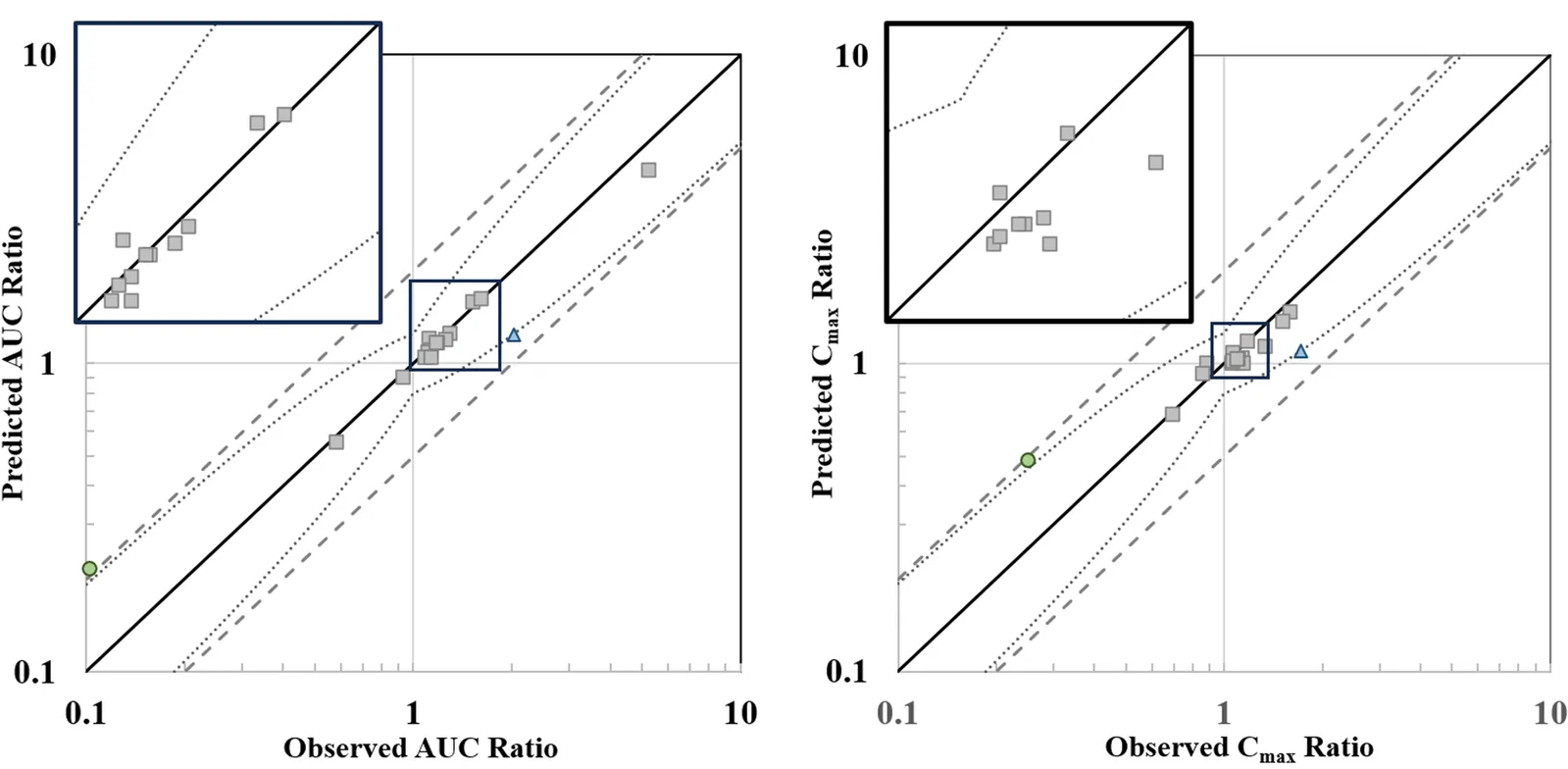
Visual PBPK model performance assessment for isavuconazole DDI studies. grey squares: predicted/observed (P/O) AUC or C_max_ ratio of study data, solid black line: P/O = 1, dotted lines: Guest criteria with 20% variability [13], dash lines: upper and lower 2-fold threshold, blue triangle: isavuconazole-midazolam (perpetrator DDI), green circle: isavuconazole-rifampin (victim DDI)

The performance of the adult isavuconazole PBPK model for both the iv and oral formulations for single and multiple dose administration can be found in Table S3 and Table 2, respectively. Model GMFE was < 1.5 for all PK parameters indicating good model performance. For isavuconazole victim DDI model performance, a summary of the predicted and observed can be found in Table S4 and perpetrator DDI in Table S5. A visual assessment of the model DDI performance can be found in Fig. 2. DDI model performance was assessed using the Guest criteria [13]. All the assessed clinical DDI studies simulated by the PBPK model fell within the Guest criteria except for the isavuconazole perpetrator DDI with midazolam and the isavuconazole victim study with rifampicin. More extensive discussion on these deviations can be found in the discussion section.

**Table 2:** Predicted and observed PK parameters for single and multiple dose isavuconazole in adults

	AUC_24_ (ng·h/mL)		Cmax (ng/mL)		C_min_ (ng/mL)	
	Predicted	Observed	Predicted	Observed	Predicted	Observed
Day 1						
100/50 mg oral	8,340 (15.7)	8,680 (14.5)	1,322 (25.1)	1,100 (15.7)		
200/100 mg oral	16,600 (16.5)	18,000 (24.6)	2,550 (25.2)	1,850 (17.9)		
80/40 mg iv	7,490 (15.0)	7,260 (15.0)	1,250 (13.2)	1,280 (9.6)		
160/80 mg iv	14,800 (15.4)	12,800 (15.2)	2,530 (13.6)	2,320 (19.2)		
Day 8						
100/50 mg oral	16,000 (36.0)	18,000 (24.6)	1,190 (21.9)	1,200 (17.9)	535 (41.9)	581 (30.1)
200/100 mg oral	32,000 (36.3)	33,900 (16.7)	2,340 (22.5)	2,330 (20.3)	1,070 (42.0)	1,156 (11.7)
80/40 mg iv	13,900 (35.2)	12,300 (30.4)	1,080 (19.2)	987 (18.3)	463 (41.0)	371 (36.9)
160/80 mg iv	27,000 (35.1)	24,400 (24.0)	2,150 (18.9)	2,520 (41.8)	895 (41.2)	752 (34.1)
Day 14						
100/50 mg oral	19,000 (41.5)	22,000 (22.3)	1,320 (26.0)	1,400 (12.7)	665 (47.3)	755 (16.7)
200/100 mg oral	37,500 (41.7)	41,400 (14.4)	2,580 (26.5)	2,610 (14.0)	1,320 (47.4)	1,680 (18.3)
80/40 mg iv†	106,000 (69.2)	83,000 (55.8)	1,190 (24.0)	1,170 (20.2)	570 (46.7)	486 (47.1)
160/80 mg iv†	209,000 (67.3)	255,000 (44.4)	2,360 (23.3)	2,550 (34.6)	1,110 (46.5)	1,053 (28.2)
**Day 21**						
**100/50 mg oral†**	141,000 (72.5)	121,000 (19.4)	1,390 (28.9)	1,370 (16.8)	736 (50.9)	725 (23.5)
**200/100 mg oral†**	267,000 (72.7)	241,000 (31.7)	2,700 (29.2)	2,560 (16.9)	1,433 (50.9)	1,450 (22.9)
**GMFE**	**1.12**		**1.08**		**1.12**	

*iv* intravenous, *GMFE* geometric mean fold error, *AUC*_*24*_ area under the curve from 0 to 24 h, *C*_*max*_ maximum concentration, *C*_*min*_ minimum or trough concentration

data presented as arithmetic mean (%coefficient of variance)

†AUC_inf_ (area under the curve from 0 to infinity) reported

The performance of the edoxaban model is detailed in the supplemental information in Tables S11 to S13 and Fig. S4. Model performance was within acceptable limits with the GMFE < 1.25 for all PK parameters and the predicted DDI fell within the Guest criteria for each study. In addition, the PBPK model effectively mimicked the observed clinical mass balance study (Table S13). Therefore, the edoxaban model was considered appropriately verified for utilization in the verification of K_i, P-gp_ for the isavuconazole PBPK model.

The performance of the pediatric isavuconazole PBPK model for both the iv and oral formulations for multiple dose administration can be found in Table 3, and in the supplemental information (Fig. S2 and S3). The GMFE for all pediatric simulations were < 1.25, indicating great model performance for both iv and oral formulations and age groups.

**Table 3:** Predicted and observed PK parameters for multiple dose isavuconazole in pediatric patients

	AUC_24_ (ng·h/mL)		C_max_ (ng/mL)		C_min_ (ng/mL)	
	Predicted	Observed	Predicted	Observed	Predicted	Observed
Day 3						
1 to < 6 years iv	109,000 (24.5)	112,000 (22.2)	8,420 (15.2)	7,810 (10.6)	3,570 (30.9)	4,200 (34.3)
6 to < 12 years iv	119,000 (25.1)	102,000 (34.5)	8,670 (15.9)	7,800 (21.0)	3,960 (30.9)	4,310 (49.1)
12 to < 18 years iv	100,000 (34.8)	70,100 (42.2)	7,310 (24.9)	5,530 (41.9)	3,410 (38.1)	2,520 (44.6)
6 to < 12 years oral	109,000 (25.9)		7,440 (17.3)		3,640 (30.7)	4,400 (39.9)
12 to < 18 years oral	88,300 (37.6)		6,110 (27.2)		3,050 (40.6)	3,250 (43.7)
Day 7						
1 to < 6 years iv	89,900 (35.4)	96,800 (48.9)	7,440 (20.2)	7,310 (16.6)	2,920 (43.5)	3,320 (51.5)
6 to < 12 years iv	101,000 (35.6)	87,200 (38.1)	7,780 (21.7)	6,780 (31.1)	3,370 (42.8)	2,970 (56.7)
12 to < 18 years iv	94,800 (40.4)	76,800 (26.6)	7,021 (27.2)	5,020 (23.8)	3,270 (45.2)	2,730 (41.6)
6 to < 12 years oral	94,600 (35.7)	111,000 (45.2)	6,740 (22.5)	6,040 (37.1)	3,180 (41.8)	3,970 (46.3)
12 to < 18 years oral	87,000 (42.2)	83,300 (40.1)	6,010 (29.2)	5,030 (43.1)	3,040 (46.1)	3,100 (52.2)
GMFE	1.16		1.17		1.17	

*iv* intravenous, *GMFE* geometric mean fold error, *AUC*_*24*_ area under the curve from 0 to 24 h, *C*_*max*_ maximum concentration, *C*_*min*_ minimum concentration

data presented as arithmetic mean (%coefficient of variance)

**Fig. 3:**
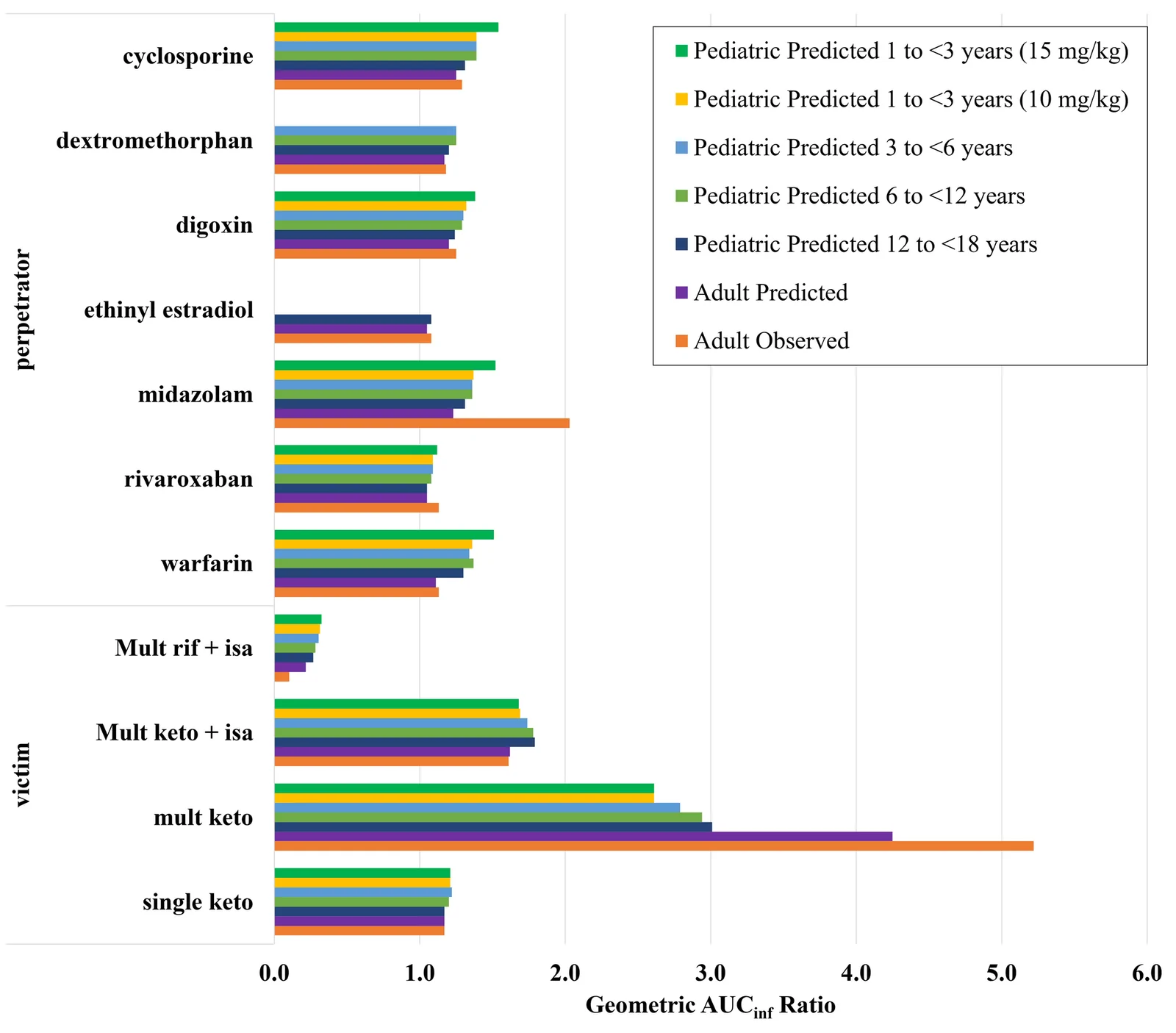
Comparison of geometric mean AUC ratio between pediatric and adult populations. keto: ketoconazole, isa: isavuconazole, mult: multiple, rif: rifampicin

The predicted geometric mean (GM) AUC_inf_ ratio between adult and pediatric populations can be found visualized in Fig. 3. Overall, the DDI risk was determined to be similar between pediatric populations and adult populations, with slightly higher DDI risk in the 1 to < 3-year-old population, especially when receiving the 15 mg/kg dose. The GM ratio values of AUC_inf_, C_max_, and their 90% confidence intervals (CI) can be found in the supplement information. The AUC_24h_ on day 14 (steady state) was assessed separately to better understand the difference in exposure between the 15 mg/kg and 10 mg/kg isavuconazonium sulfate doses in the 1 to < 3-year-old population and the 10 mg/kg dose in the 3 to < 18 years old. The GM AUC_24h_ at steady-state (day 14) was 113,000 ng·h/mL (113 mg·h/L), 78,100 ng·h/mL (78.1 mg·h/L), and 80,700 ng·h/mL (80.7 mg·h/L) for 1 to < 3 years old receiving 15 mg/kg, 1 to < 3 years old receiving 10 mg/kg, and 3 to < 18 years old receiving 10 mg/kg, respectively. Both fell above the efficacy threshold (60 mg·h/L) and below the toxicity threshold (233 mg·h/L) and agreed with the population PK model extrapolations for the 15 mg/kg dose.

Specific results from the sensitivity analysis can be found in the supplemental information (Table S6 and S7). In short, the DDI of midazolam and cyclosporine were sensitive to the values of K_i, CYP3A4_ and K_i, CYP3A5_ and the DDI of digoxin and edoxaban were not very sensitive to the value of K_i, P-gp, liver_. K_i, CYP3A5_ were less sensitive to change than K_i, CYP3A4_. Midazolam AUC ratio increased to 3.28-fold when K_i, CYP3A4_ decreased 10-fold, while only increasing to 2.05-fold when K_i, CYP3A5_ decreased 10-fold. The same trend was observed for cyclosporine AUC ratio. Digoxin and edoxaban AUC ratio increased 14% and 22%, respectively when K_i, P-gp, liver_ was decreased 10-fold.

## Discussion

### Isavuconazole PBPK model evaluation

Administering only the active substance, isavuconazole, in the PBPK model was a strategic consideration for this modeling approach. Avoiding modeling of the prodrug allowed for complex DDI simulations to be conducted and improved the resource requirements of the simulations. Administering only isavuconazole was considered acceptable because isavuconazonium sulfate is rapidly absorbed and almost completely converted to isavuconazole primarily by BChE immediately after absorption. Therefore, administering the active equivalent dose (372 mg isavuconazonium sulfate/ 200 mg isavuconazole) was considered comparable to administering the inactive prodrug. In addition, the prodrug isavuconazonium sulfate was found to have little or no inhibitory activity against CYP isozymes, except for CYP2B6 with an IC_50_ of 46.7 µmol/L (internal in vitro inhibition data), which is most likely not a clinically relevant due to the rapid conversion of the prodrug and the minimal expression of CYP2B6 in the intestinal lining.

When considering the pediatric population, generally, esterase activity (CES1/2) starts low in newborns and gradually increases [34].Studies found no significant difference in esterase IC50 values between 3-month and 36-year liver samples [35]. In another study, researchers examined BChE activity of 722 samples from infant deaths < 1 year old and compared them to healthy adult control samples. In non-Sudden Infant Death Syndrome (SIDS) samples, the BChE activity was comparable to the adult control samples [36]. Although this study focused on how BChE levels may predict the occurrence of SIDS, the study also indicates that BChE activity in pediatric patients > 1 year should be comparable to adults. Considering that isavuconazole is approved for patients > 1 year-old, we concluded that isavuconazonium sulfate conversion would not be significantly altered in pediatric populations when compared to adults. Therefore, accounting for the inactive prodrug in the PBPK model was not required for the present application. Any potential differences in BChE activity may lead to decreased C_max_ and increase t_max_; however, the impact of this difference on the overall predicted DDI should be minimal.

The model GMFE for all PBPK models was < 1.5 for all reported PK parameters and < 1.25 for all but AUC in the adult single dose iv isavuconazole studies, which was influenced by intersubject variability in the small subject number single ascending dose iv study. In the study design for this trial, different subjects (*n* = 6) were enrolled for each dosing cohort; therefore, it is possible that the observed difference in CL between each cohort might be from intersubject variability and the low sample number. In addition, the PopPK analysis found isavuconazole PK to be linear and dose-proportional with interpatient variability around 60% [22].

The model captured all observed DDI studies well except for the multiple-dose rifampicin (isavuconazole as victim) and multiple-dose isavuconazole with midazolam (isavuconazole as perpetrator) studies. The reason for model under prediction of the DDI magnitude for both studies was unclear; however, several hypotheses were explored. For the rifampicin study, it was hypothesized that the magnitude of induction utilized within the rifampicin PBPK did not reflect the clinical trial data. Another potential reason for the under prediction might stem from UGT induction, rifampicin is an inducer of UGT [37] and UGT was not included in the isavuconazole model. An exploratory analysis (data not shown) was conducted where f_m, CYP3A4/5_ in the isavuconazole model and percent CYP3A5 EM in the virtual population was explored. No combination of values was identified that recovered the observed AUC ratio. Only increasing the induction parameters (Ind_max_ and IndC_50_) within the rifampicin compound file improved the predicted AUC ratio.

Another factor to consider is the inter-patient variability associated with CYP induction. Generally, interpatient variability in CYP induction is much larger than with CYP inhibition due to the mechanism of CYP induction (i.e. increase in enzyme abundance through increased expression). Simcyp reported verification of rifampicin PBPK compound file induction covered 74 different observed studies; however, model performance across all studies varied. Most studies fell within the < 1.5-fold predicted/observed (P/O) ratio, but several studies over (P/O < 0.5-fold) or under (P/O > 2-fold) predicted the induction effect. Rifampicin model performance was not perfect across all studies and may indicate factors not fully captured by the rifampicin model. Therefore, it is possible that CYP3A inhibition of isavuconazole is well captured by the models, but CYP3A induction may not be. The best practice approach assesses overall model performance across multiple studies, selecting values that are based on in vitro data and/or utilizing an optimized parameter that accurately captures most study data. Therefore, considering that the isavuconazole PBPK model captured all three ketoconazole DDI studies well, each study was conducted with different subjects and different dosing regimens, and altering the f_m, CYP3A4/5_ had no impact on the simulated AUC ratio, it was determined that the f_m, CYP3A_ utilized within the model was appropriate for the present application and any uncertainty would have minimal impact on the predicted DDI in pediatric populations.

For midazolam, while the predicted AUC and C_max_ ratios fall just outside the Guest criteria, it still falls within the 2-fold error threshold. The possible rationales for the underprediction are as follows: (1) because midazolam is also metabolized by UGT, and isavuconazole was found to have potential to inhibit UGT, (2) CYP3A4 vs. CYP3A5 metabolism, and (3) First-pass gut metabolism plays a significant role in the PK of midazolam. First, UGT metabolism. The major metabolite of midazolam, 1-OH-midazolam, is primarily eliminated via UGT and GM AUC_inf_ of 1-OH-midazolam in the observed clinical DDI study increased by 25% when midazolam was coadministered with isavuconazole [38]. However, UGT inhibition could not be incorporated into the model due to the lack of specific data to optimize the different UGT isoforms. Second, CYP3A4 versus CYP3A5 metabolism. There could have been a difference in the frequency of CYP3A5 extensive metabolizers between the observed population and the virtual population. However, since CYP3A5 expression information was not captured in clinical trials, this variable could not be explored. It is possible that isavuconazole inhibits CYP3A5 to a greater degree than CYP3A4; however, without data to support this conclusion it was difficult to determine. The model assumed 83% CYP3A5 null expressors for all simulations where phenotype distribution was unknown. This is important since midazolam clearance can be 1.7-fold higher in CYP3A5 expressors [39]. Finally, the bioavailability of oral midazolam is highly variable ranging from 30 to 72% due to high first-pass metabolism and sometimes incomplete oral absorption [40]. Both factors seem to be supported by the fact that the 90% CI for GM AUC_inf_ ratio was large. In addition, CYP3A4 and CYP3A5 are both present within the GI tract and mucosal CYP3A is a significant contributor to the first-pass metabolism of midazolam [41].

A final rationale for both the midazolam and rifampicin studies might be the impact of EHR. The isavuconazole PBPK model used first-order absorption without EHR. When EHR occurs, contribution of hepatic metabolism may increase as the reabsorbed drug is circulated back to the liver via the portal vein. The ADAM model is required to model EHR; however, model performance decreased significantly when the ADAM model was implemented, since in the PBPK model, isavuconazole was administered instead of isavuconazonium sulfate. The lack of EHR within the isavuconazole model might be a contributing factor for both midazolam and rifampicin under prediction. Further research may be needed to fully understand the impact of isavuconazole EHR on intestinal CYP3A.

For completion, the K_i_ required to capture the midazolam study was explored. A K_i, CYP3A4/5_ value of 0.08 µM was required to capture the observed midazolam study. When this value was utilized in the cyclosporine studies, the PBPK model overpredicted the cyclosporine AUC ratio. When a sensitivity analysis was performed on K_i, CYP3A4_ and K_i_,_CYP3A5_, no combination of values were identified that could capture the observed DDI from both the midazolam and the cyclosporine studies (supplemental Table S6). Since there were two cyclosporine studies with different subjects and clinical trial design (dose and schedule), K_i_ values were preferentially optimized to one cyclosporine study and then verified with the second cyclosporine study. In addition, there was agreement between the observed and predicted GM AUC_inf_ ratio for other CYP3A4 substrates: ethinyl estradiol, rivaroxaban and edoxaban. The K_i_ and/or Ind_max_ values of CYP3A4, CYP3A5, and P-gp for all PBPK models were considered well verified. The multiple observed clinical DDI studies affecting these enzymes and/or transporters allowed for optimization and subsequent verification on different datasets.

### Model limitations

Not all inhibition (K_i_) and induction (Ind_max_ and Ind_C50_) parameters for the isavuconazole PBPK could be fully verified, but for potential future model applications, other CYP enzymes and transporters that are impacted by isavuconazole were still included except for MATE1, OCT1, OCT2, and OATP1B1. It is also important to note that optimization for the K_i_ values for CYP2C8 and CYP2C19 could not be adequately performed; therefore, the parameters were left as the in vitro measured values. However, it was hypothesized based on observed clinical trials, that isavuconazole does not impact CYP2C8 and CYP2C19 to a clinically significant degree. The K_i_ values for CYP3A4 and CYP3A5 were assumed to be the same based on the reported in vitro values. The K_i_ for P-gp was assumed to be the same in all tissues where the transporter is located (e.g., intestines, liver, kidney). For the present application, CYP3A and P-gp inhibition parameters were considered sufficiently verified for the intended purpose; however, additional verification may be needed for the other CYP enzymes, or the transporters not included in the model.

Initial model construction and verification were done in healthy adult subjects while the DDI prediction was performed in pediatric cancer patients. Additional verification of CYP and transporter inhibition/induction parameters could not be performed in pediatric cancer patients due to the lack of available data. It is reasonable to assume that these values will not change significantly based on the population and are instead innate to the compound. This conclusion is further substantiated by the definition of a K_i_ value, which is the dissociation constant describing the binding affinity between the inhibitor and the enzyme. Therefore, unless the binding pocket of the given enzyme changes with the population, it is unlikely that population would affect the model determined K_i_ value.

Finally, the pediatric population model provided within the simulator uses adult anatomical and physiological data for the permeability-limited liver model. It is unknown if changes need to be made to adjust the permeability-limited liver model from adults to pediatric participants. This difference could affect P-gp transport; however, P-gp behavior in other pediatric PBPK models have seemed appropriate to continue this approach [42–44] .

Overall, the sensitivity analysis indicated that K_i_ values were sensitive and could change the model outcomes. There was good confidence in the K_i, CYP3A4_ used in isavuconazole model due to the considerable number of DDI studies with CYP3A4 involvement utilized during model development. For P-gp, there were less full clinical DDI studies available which garnered less confidence. However, considering that the model was only marginally sensitive to a 10-fold decrease in K_i, P-gp_, the confidence in the relative DDI risk reported herein increases. Based on the sensitivity analysis, it appears that P-gp inhibition by isavuconazole might be close to the clinically maximal inhibitory effect.

### Prediction of DDI risk in pediatric population

Overall, the model predicted DDI risk was similar between adult and the pediatric populations. There was a trend of greater isavuconazole perpetrator DDI risk in the 1 to < 3-year-old pediatric population receiving the 15 mg/kg dose with a predicted GM AUC_inf_ ratio 7 to 36% greater than predicted in adults. This trend is consistent with literature reported observations, with greater DDI risk in younger pediatric subjects, although that observation was not consistent across all medications [45]. Based on the analysis of 24 DDI drug pair reports conducted by Salem et al., the increased DDI risk observed in patients < 2 years old and the vulnerability of this population warrants extra care. In the present analysis, the increased risk observed in the 1 to < 3-year-old population receiving the 15 mg/kg dose appears to be due to the higher dose, as the population receiving the 10 mg/kg dose had similar DDI risk to the other pediatric populations. However, as mentioned in the results section, the 1 to < 3 years old population receiving the 10 mg/kg dose had a slightly lower day 14 AUC_24h_ than the > 3 years old population with the same dose, likely due to differences in CYP abundance. Therefore, in clinical practice, it is important to consider which dose of isavuconazonium sulfate is being administered when assessing DDI risk potential.

The 15 mg/kg isavuconazonium sulfate dose was implemented by the FDA for subjects 1 to < 3 years old to account for increased CYP3A activity in children compared to adults. In addition, steady state exposure at the 10 mg/kg dose in 1 to < 3 years was found to be lower than the other age ranges and close to the minimum therapeutic exposure. However, this dose was never tested clinically and the number of subjects < 3 years of age in the clinical trial was low (*n* = 5). However, due to the high toxicity threshold (233 mg·h/L), the cautionary increased starting dose is reasonable. The model predicted isavuconazole AUC_24h_ at steady state (day 14) was ~ 40% higher in the 1 to < 3-year-old (15 mg/kg) cohort versus the 3 to < 18-year-old (10 mg/kg) cohort but was still within the established AUC_24h_ therapeutic range. Unfortunately, PBPK model performance at 15 mg/kg could not be verified; therefore, it cannot be ruled out that the PBPK model overpredicts the exposure in this population at this dose. If the model was overpredicting the exposure, then the model predicted DDI represents a worst-case scenario, which is preferred. Interestingly, when analyzing the isavuconazole victim DDI risk (strong CYP3A inhibitor or inducer), the DDI risk was lower in the pediatric population than the adult population. This may be due to the increased CYP3A activity seen in pediatric populations until adolescence.

It is important to consider that the DDI with midazolam and rifampicin were underestimated in the adult model; therefore, it is highly possible that the predicted pediatric risk may be underestimated as well. When considering midazolam, oral bioavailability is just as variable in pediatric populations (9%-71%) as observed in adults, even more so due to the variability in the maturity of CYP3A intestinal and liver metabolism [46]. Therefore, caution should be implemented when co-administering midazolam and isavuconazole in a pediatric population. The same caution should be implemented when considering narrow therapeutic window drugs such as warfarin and digoxin, which both had predicted AUC ratios > 1.5-fold.

## Conclusions

We reported the development and verification of an isavuconazole PBPK model for application in adult and pediatric populations. The verified PBPK model was then utilized to predict the potential difference in DDI risk in pediatric populations when compared to the observed clinically conducted studies and model predicted in adults. We observed minimal differences in the magnitude of DDI between adult and pediatric populations despite the difference in CYP activity. This observation is likely due to the difference in doses utilized between adults and children which accounts for the difference in size (weight height) and metabolic potential. There appears to be a trend of higher perpetrator DDI risk in children 1 to < 3 years of age receiving the 15 mg/kg dose due to the greater steady state isavuconazole exposure. This report represents another application of PBPK to fill gaps in clinical knowledge leading to better clinical understanding of therapeutic interventions. Although additional studies may be needed to fully verify all enzyme and transporter DDI mediated by isavuconazole, the developed isavuconazole adult and pediatric models may now be utilized to predict DDI risk with other potential CYP3A and P-gp inhibitors and substrates with high confidence.

## Supplementary Information

Below is the link to the electronic supplementary material.

Supplementary Material 1

## Data Availability

Details for how researchers may request access to anonymized participant level data, trial level data and protocols from Astellas sponsored clinical trials can be found at https://www.clinicaltrials.astellas.com/transparency/.
