# Supplementary material for: Development of an isavuconazole physiologically-based pharmacokinetic model for adult and pediatric populations
Source: J Pharmacokinet Pharmacodyn. 2026 Sep 22;53(6):51. doi: 10.1007/s10928-026-10061-8 (PMC13597547; doi:10.1007/s10928-026-10061-8)
Supplement: Supplementary file 1 [file 10928_2026_10061_MOESM1_ESM.docx]

**Development of an Isavuconazole Physiologically-based Pharmacokinetic Model for Adult and Pediatric Populations**

Mary P Choules, PharmD, PhD, Amit Desai, PhD, Laura Lynn Kovanda, PhD, Yukio Otsuka, PhD, Roger Brüggemann, PhD, and Peter L Bonate, PhD

# Isavuconazole

When the model was translated to pediatrics, all drug model parameters remained the same as the adult model. The CL_int_ for CYP3A4 and CYP3A5 that was calculated for the adult model remained the same for the pediatric model. Altering CL_int_ was not required since simulated total clearance is dependent on the population CYP abundance for each simulated patient. For the pediatric simulations, the designated ontogeny (discussed more in the Population model section) drives the virtual patient CYP abundance. Of note, due to the clinical trial design, participants were allowed to receive their first maintenance dose (starting on day 3 of dosing) anywhere from 12 to 24 hours following their last loading dose. Therefore, participants were split into two categories based on if they receive their first maintenance dose 12 or 24 hours following the last loading dose. PK parameters were summarized based on a combination of these categories while observed versus simulated plasma concentration-time curves remain separated.

Fig. S1	Mass balance diagram of the prodrug isavuconazonium sulfate

Table S1	Summary of PK studies used in the development and verification of the isavuconazole PBPK model

| **Study (reference)** | **Dose** | **Form** | **Cohort** | **N** | **Age**  **(years)** | **Weight**  **(kg)** |
| --- | --- | --- | --- | --- | --- | --- |
| adult SAD(13) | 100, 200, 400 mg | oral | healthy males | 15 | 22-45 | 57-88 |
| adult SAD(13) | 40, 80, 160 mg | iv | healthy males | 18 | 23-45 | 57-89 |
| adult MAD(14) | day 1: 100 / 200 mg  day 2-14: 50 / 100 mg | oral | healthy males | 12 | 21-45 | 66-94 |
| adult MAD(14) | day 1: 80 / 160 mg  day 2-14: 40 / 80 mg | iv | healthy males | 12 | 26-45 | 65-101 |
| pediatric(15) | 10 mg/kg (200 mg max)  day 1: Q8H  day3-28: daily | iv | children requiring antifungal prophylaxis | 26 | 1 to <18 | 9-104 |
| pediatric(15) | 10 mg/kg (200 mg max)  day 1: Q8H  day3-28: daily | oral | children requiring antifungal prophylaxis | 19 | 6 to <18 | 18-93 |

SAD: single ascending dose, MAD: multiple ascending dose, Q8H: every 8 hours

Table S2	Summary of DDI studies used in the development and verification of the isavuconazole PBPK model

| **Perpetrator** | **Perpetrator Dosing** | **Victim** | **Victim Dosing** | **Cohort** | **N** | **Age (yr)** | **Ref** |
| --- | --- | --- | --- | --- | --- | --- | --- |
| ketoconazole | day 1: 200 mg | isavuconazole | day 1: 400 mg | healthy males | 12 | 21-45 | (9) |
| ketoconazole | day 1-24: 200 mg Q12H | isavuconazole | day 4: 200 mg | Healthy  (0.33 female) | 12 | 19-53 | (9) |
| ketoconazole | day 1-36: 200 mg QD | isavuconazole | day 9: 400 mg day 10-22: 100 mg (2h lag) | healthy males | 26 | 19-64 | (9) |
| rifampicin | day 1-36: 600 mg QD | isavuconazole | day 9: 400 mg day 10-22: 100 mg (2h lag) | healthy males | 26 | 19-64 | (9) |
| isavuconazole | day 1: 400 mg day 2-28: 100 mg | warfarin | day 21: 10 mg (2h lag) | healthy males | 12 | 22-45 | (16) |
| isavuconazole | day 1-2: 200mg Q8H day 3-13: 200 mg QD | warfarin | day 5: 20 mg | healthy males | 20 | 23-54 | (16) |
| isavuconazole | day 1: 400 mg day 2-20: 100 mg QD | cyclosporine | day 15: 300 mg | healthy males | 26 | 22-43 | (17) |
| isavuconazole | day 1-2: 200mg Q8H day 3-8: 200 mg QD | cyclosporine | day 5: 300 mg | Healthy  (0.21 female) | 24 | 18-54 | (17) |
| isavuconazole | day 1-2: 200 mg Q8H day 3-11: 200 mg QD | midazolam | day 10: 3 mg | Healthy  (0.3 female) | 23 | 19-54 | (9) |
| isavuconazole | day 1-2: 200 mg Q8H day 3-12: 200 mg QD | digoxin | day 5: 0.5 mg | Healthy  (0.25 female) | 24 | 20-55 | (18) |
| isavuconazole | day 1-2: 200 mg Q8H day 3-7: 200 mg QD | dextromethorphan | day 5: 30 mg | Healthy  (0.29 female) | 24 | 21-53 | (19) |
| isavuconazole | day 1-2: 200 mg Q8H day 3-13: 200 mg daily | repaglinide | day 10: 0.5 mg | healthy males | 24 | 24-52 | (19) |
| isavuconazole | day 1-2: 200 mg Q8H day 3-8: 200 mg daily | ethinyl estradiol | day 5: 0.035 mg | healthy females | 24 | 51-65 | (9) |
| isavuconazole | day 1-2: 200 mg Q8H day 3-13: 200 mg daily | bupropion | day 8: 100 mg | Healthy  (0.46 female) | 24 | 21-55 | (19) |
| Isavuconazole | day 1: 200 mg Q8H | rivaroxaban | day 1: 25 µg 1 hr after | Healthy  (0.42 female) | 12 | 20-48 | (20) |
| isavuconazole | day 1: 200 mg Q8H | edoxaban | day 1: 50 µg 1 hr after | Healthy  (0.42 female) | 12 | 20-48 | (20) |

yr: year, QXH: every X hours, QD: every day

Table S3	Predicted and observed PK parameters for single dose isavuconazole in adults

|  | **AUC_inf_ (ng·h/mL)** | | **C_max_ (ng/mL)** | | | **CL (L/h)** | | |  |
| --- | --- | --- | --- | --- | --- | --- | --- | --- | --- |
|  | **Predicted** | **Observed** | **Predicted** | | **Observed** | **Predicted** | | **Observed** |  |
| **100 mg oral** | 44,600 (50.8) | 36,700 (18.4) | 1,290 (24.9) | | 1,450 (12.2) | 2.89 (55.6) | | 2.81 (19.6) |  |
| **200 mg oral** | 89,200 (49.5) | 77,900 (13.7) | 2,560 (26.5) | | 2,590 (17.4) | 2.86 (55.2) | | 2.61 (14.0) |  |
| **400 mg oral** | 183,000 (56.4) | 211,000 (19.8) | 5,680 (24.0) | | 5,570 (3.8) | 2.74 (43.9) | | 1.96 (22.2) |  |
| **40 mg iv** | 19,100 (49.6) | 11,500 (41.9) | 632 (13.3) | | 446 (17.1) | 2.64 (51.6) | | 4.04 (41.9) |  |
| **80 mg iv** | 38,300 (49.7) | 26,200 (24.3) | 1,270 (13.3) | | 1,030 (17.9) | 2.64 (51.6) | | 3.22 (26.9) |  |
| **160 mg iv** | 77,100 (49.4) | 72,100 (17.8) | 2,530 (13.4) | | 2,470 (15.1) | 2.62 (51.6) | | 2.28 (19.4) |  |
| **GMFE** | **1.27** | | | **1.13** | | | **1.22** | |  |

iv: intravenous, GMFE: geometric mean fold error, AUC_inf_: area under the curve from 0 to infinity, C_max_ maximum concentration, CL: total clearance (dose/AUC_inf_)

CL/F reported for oral formulations

data presented as arithmetic mean (%coefficient of variance)

Table S4	Summary of predicted by the PBPK model and observed PK parameters (AUC and C_max_ ratio) of DDI for isavuconazole as the victim drug

|  | **Without Inhibitor** | | **With Inhibitor** | |  | |
| --- | --- | --- | --- | --- | --- | --- |
|  | **^‡^AUC_inf_ (ng*h/mL)** | **C_max_ (ng/mL)** | **^‡^AUC_inf_ (ng*h/mL)** | **C_max_ (ng/mL)** | **^‡^AUC_inf_ ratio** | **C_max_ ratio** |
| **Single dose 200 mg ketoconazole** | | | | | | |
| **Observed** | 154,000 | 4,260 | 181,000 | 4,690 | **1.17** | **1.10** |
| **90% CI** |  |  |  |  | (1.10, 1.25) | (0.993, 1.22) |
| **Predicted** | 162,000 | 4,950 | 190,000 | 5,170 | **1.17** | **1.04** |
| **90% CI** |  |  |  |  | (1.16, 1.18) | (1.04, 1.05) |
| **Criteria** |  |  |  |  | **0.861 - 1.59** | **0.834 - 1.45** |
| **Multiple dose 200 mg ketoconazole BID** | | | | | | |
| **Observed** | 82,309 | 2,180 | 430,000 | 2,380 | **5.22** | **1.09** |
| **90% CI** |  |  |  |  | (4.09, 6.66) | (0.933, 1.27) |
| **Predicted** | 82,500 | 2,514 | 351,000 | 2,630 | **4.25** | **1.04** |
| **90% CI** |  |  |  |  | (4.01, 4.50) | (1.04, 1.05) |
| **Criteria** |  |  |  |  | **2.81 - 9.69** | **0.831 - 1.43** |
| **Multiple dose 200 mg ketoconazole daily + multiple dose isavuconazole** | | | | | | |
| **Observed** | 42,600‡ | 2,730 | 68,800‡ | 4,130 | **1.61**‡ | **1.51** |
| **90% CI** |  |  |  |  | (1.50, 1.74) | (1.39, 1.64) |
| **Predicted** | 34,700‡ | 2,460 | 56,300‡ | 3,400 | **1.62**‡ | **1.38** |
| **90% CI** |  |  |  |  | (1.59, 1.66) | (1.36, 1.40) |
| **Criteria** |  |  |  |  | **1.05 - 2.47** | **1.00 - 2.27** |
| **Multiple dose 600 mg rifampicin daily + multiple dose isavuconazole** | | | | | | |
| **Observed** | 37,100‡ | 2,340 | 3,770‡ | 584 | **0.102**‡ | **0.250** |
| **90% CI** |  |  |  |  | (0.0936, 0.110) | (0.230, 0.271) |
| **Predicted** | 34,700‡ | 2,453 | 7,480‡ | 1,191 | **0.216**‡ | **0.485** |
| **90% CI** |  |  |  |  | (0.208, 0.224) | (0.477, 0.494) |
| **Criteria** |  |  |  |  | **0.0530 - 0.196** | **0.138 - 0.453** |

AUC_inf_: area under the curve from 0 to infinity, C_max_: maximum concentration, CI: confidence interval

AUC ratio: AUC(with inhibitor)/AUC(without inhibitor), C_max_ ratio: C_max_(with inhibitor)/C_max_(without inhibitor)

Criteria determined by GUEST method assuming 20% variability in pharmacokinetic parameters(21)

‡Multiple dose studies reported as AUC_24h_

Table S5	Summary of Predicted by the PBPK Model and Observed PK parameters (AUC and C_max_ ratio) of DDI for Isavuconazole as the Perpetrator

|  | **Without Isavuconazole** | | **With Isavuconazole** | |  | |
| --- | --- | --- | --- | --- | --- | --- |
|  | **AUC_inf_ (ng*h/mL)** | **C_max_**  **(ng/mL)** | **AUC_inf_ (ng*h/mL)** | **C_max_**  **(ng/mL)** | **AUC_inf_ ratio** | **C_max_ ratio** |
| **S Warfarin 10 mg (20 mg Warfarin) - *CYP2C9** | | | | | | |
| **Observed** | 37,500 | 1,110 | 41,500 | 978 | **1.11** | **0.884** |
| **90% CI** |  |  |  |  | (1.06, 1.16) | (1.06, 1.21) |
| **Predicted** | 38,600 | 873 | 46,800 | 886 | **1.21** | **1.01** |
| **90% CI** |  |  |  |  | (1.21, 1.22) | (1.01, 1.03) |
| **Criteria** |  |  |  |  | **0.838 - 1.47** | **0.661 - 1.18** |
| **S Warfarin 5 mg (10 mg Warfarin) - CYP2C9** | | | | | | |
| **Observed** | 17,100 | 628 | 19,300 | 658 | **1.13** | **1.05** |
| **90% CI** |  |  |  |  | (1.03, 1.24) | (0.911, 1.20) |
| **Predicted** | 19,300 | 483 | 21,400 | 487 | **1.11** | **1.01** |
| **90% CI** |  |  |  |  | (1.10, 1.12) | (1.01, 1.01) |
| **Criteria** |  |  |  |  | **0.846 - 1.51** | **0.817 - 1.35** |
| **Cyclosporine 300 mg (study 1) - *CYP3A4, CYP3A5** | | | | | | |
| **Observed** | 6,660 | 1,360 | 7,330 | 1,490 | **1.10** | **1.09** |
| **90% CI** |  |  |  |  | (0.996 - 1.22) | (0.990 - 1.20) |
| **Predicted** | 6,420 | 1,800 | 7,260 | 1,890 | **1.13** | **1.05** |
| **90% CI** |  |  |  |  | (1.13, 1.13) | (1.05, 1.05) |
| **Criteria** |  |  |  |  | **0.834 - 1.45** | **0.831 - 1.43** |
| **Cyclosporine 300 mg (study 2) - CYP3A4, CYP3A5** | | | | | | |
| **Observed** | 7,300 | 1,320 | 9,390 | 1,400 | **1.29** | **1.06** |
| **90% CI** |  |  |  |  | (1.15, 1.44) | (0.953, 1.19) |
| **Predicted** | 6,480 | 1,820 | 8,170 | 1,990 | **1.25** | **1.09** |
| **90% CI** |  |  |  |  | (1.24, 1.26) | (1.09, 1.10) |
| **Criteria** |  |  |  |  | **0.909 - 1.83** | **0.820- 1.37** |
| **Midazolam 3 mg - CYP3A4, CYP3A5, UGT** | | | | | | |
| **Observed** | 43.8 | 9.99 | 88.9 | 17.2 | **2.03** | **1.72** |
| **90% CI** |  |  |  |  | (1.73, 2.38) | (1.44, 2.05) |
| **Predicted** | 33.5 | 10.1 | 41.3 | 11.0 | **1.23** | **1.09** |
| **90% CI** |  |  |  |  | (1.22, 1.24) | (1.08, 1.10) |
| **Criteria** |  |  |  |  | **1.24 - 3.31** | **1.10 - 2.69** |
| **Digoxin 0.5 mg - P-gp** | | | | | | |
| **Observed** | 32.8 | 1.54 | 41.1 | 2.04 | **1.25** | **1.33** |
| **90% CI** |  |  |  |  | (1.17, 1.34) | (1.19, 1.49) |
| **Predicted** | 27.3 | 2.09 | 32.8 | 2.39 | **1.20** | **1.14** |
| **90% CI** |  |  |  |  | (1.19, 1.21) | (1.14, 1.15) |
| **Criteria** |  |  |  |  | **0.893 - 1.75** | **0.926 - 1.91** |
| **Dextromethorphan 30 mg - *CYP2D6, CYP2B6, CYP2C9, CYP2C18, CYP2C19, CYP3A4** | | | | | | |
| **Observed** | 18.58 | 2.00 | 21.8 | 2.35 | **1.18** | **1.17** |
| **90% CI** |  |  |  |  | (1.02, 1.35) | (1.02, 1.35) |
| **Predicted** | 29.6 | 3.01 | 34.7 | 3.59 | **1.17** | **1.19** |
| **90% CI** |  |  |  |  | (1.15, 1.19) | (1.18, 1.21) |
| **Criteria** |  |  |  |  | **0.865 - 1.61** | **0.861 - 1.59** |
| **Repaglinide 0.5 mg - *CYP2C8, CYP3A4** | | | | | | |
| **Observed** | 10.3 | 7.96 | 9.54 | 6.83 | **0.923** | **0.858** |
| **90% CI** |  |  |  |  | (0.855, 0.997) | (0.794, 0.927) |
| **Predicted** | 9.01 | 6.93 | 8.16 | 6.44 | **0.91** | **0.929** |
| **90% CI** |  |  |  |  | (0.897, 0.914) | (0.923, 0.935) |
| **Criteria** |  |  |  |  | **0.706 - 1.21** | **0.633 - 1.16** |
| **Ethinyl estradiol 35 µg - CYP3A4, CYP1A2, CYP2C9, CYP2C8** | | | | | | |
| **Observed** | 1.02 | 0.0870 | 1.11 | 0.0991 | **1.08** | **1.14** |
| **90% CI** |  |  |  |  | (1.03, 1.13) | (1.03, 1.26) |
| **Predicted** | 1.08 | 0.0712 | 1.13 | 0.0719 | **1.05** | **1.01** |
| **90% CI** |  |  |  |  | (1.05, 1.05) | (1.01, 1.01) |
| **Criteria** |  |  |  |  | **0.827 - 1.41** | **0.849 - 1.53** |
| **Bupropion 100 mg - *CYP2B6, CYP3A4, CYP2C19** | | | | | | |
| **Observed** | 686 | 140 | 397 | 96.8 | **0.578** | **0.690** |
| **90% CI** |  |  |  |  | (0.521, 0.641) | (0.618, 0.771) |
| **Predicted** | 664 | 119 | 370 | 82.1 | **0.557** | **0.689** |
| **90% CI** |  |  |  |  | (0.540, 0.574) | (0.672, 0.706) |
| **Criteria** |  |  |  |  | **0.369 - 0.905** | **0.465 - 1.02** |
| **Rivaroxaban 20 µg - CYP3A4, P-gp** | | | | | | |
| **Observed** | 3.63 | 0.822 | 4.12 | 0.871 | **1.13** | **1.06** |
| **90% CI** |  |  |  |  | NR | NR |
| **Predicted** | 2.10 |  | 2.21 |  | **1.05** | **1.02** |
| **90% CI** |  |  |  |  | (1.05, 1.06) | (1.00, 1.06) |
| **Criteria** |  |  |  |  | **0.846 - 1.51** | **0.820 - 1.37** |
| **Edoxaban 50 µg - CYP3A4, P-gp** | | | | | | |
| **Observed** | 0.862 | 0.132 | 1.31 | 0.210 | **1.51** | **1.59** |
| **90% CI** |  |  |  |  | NR | NR |
| **Predicted** | 1.52 | 0.253 | 2.41 | 0.374 | **1.59** | **1.48** |
| **90% CI** |  |  |  |  | (1.54, 1.63) | (1.43, 1.53) |
| **Criteria** |  |  |  |  | **1.00 - 2.27** | **1.04 - 2.43** |

AUC_inf_: area under the curve from 0 to infinity, C_max_: maximum concentration, CI: confidence interval

AUC ratio: AUC(with inhibitor)/AUC(without inhibitor), C_max_ ratio: C_max_(with inhibitor)/C_max_(without inhibitor)

Criteria determined by GUEST method assuming 20% variability in pharmacokinetic parameters(21)

*Enzyme or transporter in red optimized with the indicated simulation

Fig. S2	Plasma concentration time curves of the observed and simulated multiple iv dose isavuconazole in pediatric patients

**1 to <6 years old**

**24 hr**

**12 hr**

**6 to <12 years old**

**24 hr**

**12 hr**

**12 to <18 years old**

**24 hr**

**12 hr**

12hr/24h: received first maintenance dose 12 or 24 hours after the last loading dose

Black circles: observed clinical data, solid black line: simulated arithmetic mean, dark grey line: simulated 95th percentile, light grey line: simulated 5th percentile

Fig. S3	Plasma concentration time curves of the observed and simulated multiple oral dose isavuconazole in pediatric patients

**6 to <12 years old**

**12 hr**

**24 hr**

**12 to <18 years old**

**12 hr**

**24 hr**

12hr/24h: received first maintenance dose 12 or 24 hours after the last loading dose

Black circles: observed clinical data, solid black line: simulated arithmetic mean, dark grey line: simulated 95th percentile, light grey line: simulated 5th percentile

Table S6	Sensitivity analysis for isavuconazole K_i,CYP3A4/5_

|  | **Midazolam** | | | | **Cyclosporine** | | | |
| --- | --- | --- | --- | --- | --- | --- | --- | --- |
|  | **K_i,CYP3A4_** | | **K_i,CYP3A5_** | | **K_i,CYP3A4_** | | **K_i,CYP3A5_** | |
| **K_i,CYP3A_**  **(µmol/L)** | **AUC ratio** | **C_max_ ratio** | **AUC ratio** | **C_max_ ratio** | **AUC ratio** | **C_max_ Ratio** | **AUC ratio** | **C_max_ Ratio** |
| 0.019 | 3.28 | 1.59 | 2.05 | 1.48 | 3.45 | 1.51 | 2.14 | 1.45 |
| 0.032 | 2.40 | 1.40 | 1.78 | 1.35 | 2.51 | 1.39 | 1.87 | 1.35 |
| 0.053 | 1.85 | 1.26 | 1.55 | 1.24 | 1.92 | 1.28 | 1.63 | 1.25 |
| 0.088 | 1.50 | 1.15 | 1.38 | 1.16 | 1.55 | 1.18 | 1.45 | 1.16 |
| 0.15 | 1.28 | 1.08 | 1.26 | 1.10 | 1.32 | 1.11 | 1.31 | 1.09 |
| 0.25 | 1.14 | 1.04 | 1.17 | 1.06 | 1.17 | 1.05 | 1.22 | 1.06 |
| 0.41 | 1.06 | 1.01 | 1.12 | 1.04 | 1.08 | 1.02 | 1.15 | 1.04 |
| 0.68 | 1.01 | 0.991 | 1.08 | 1.02 | 1.03 | 0.996 | 1.11 | 1.02 |
| 1.14 | 0.981 | 0.980 | 1.06 | 1.01 | 0.992 | 0.988 | 1.09 | 1.02 |
| 1.90 | 0.962 | 0.973 | 1.04 | 1.01 | 0.971 | 0.983 | 1.07 | 1.01 |

PM: poor metabolizer, EM: extensive metabolizer, ISA: isavuconazole, POSA: posaconazole

Values above and below the thick black line are respectively 10-fold lower and higher than the final model value

Highlighted rows represent the closest values to the observed AUC and C_max_ ratio for the respective studies

*Sensitivity analysis for K_i,CYP3A5_ was carried out in a CYP3A5 extensive metabolizer

Table S7	Sensitivity analysis for isavuconazole K_i,P-gp,liver_

|  | **Digoxin** | | **Edoxaban** | |
| --- | --- | --- | --- | --- |
| **K_i,P-gp,liver_**  **(µmol/L)** | **AUC ratio** | **C_max_ ratio** | **AUC ratio** | **C_max_ Ratio** |
| 0.0010 | 1.39 | 1.28 | 2.07 | 1.75 |
| 0.0017 | 1.36 | 1.25 | 2.01 | 1.74 |
| 0.0028 | 1.32 | 1.22 | 1.93 | 1.73 |
| 0.0046 | 1.27 | 1.18 | 1.82 | 1.70 |
| 0.0077 | 1.22 | 1.14 | 1.70 | 1.67 |
| 0.013 | 1.18 | 1.11 | 1.57 | 1.62 |
| 0.022 | 1.14 | 1.08 | 1.44 | 1.55 |
| 0.036 | 1.11 | 1.06 | 1.32 | 1.46 |
| 0.060 | 1.09 | 1.05 | 1.23 | 1.37 |
| 0.100 | 1.08 | 1.04 | 1.16 | 1.29 |

Values above and below the thick black line are respectively 10-fold lower and higher than the final model value

Table S8	PBPK predicted DDI in pediatric population vs adult population

| **AUC Ratio** | | | | | | | | | | | | | | | | | | |  |
| --- | --- | --- | --- | --- | --- | --- | --- | --- | --- | --- | --- | --- | --- | --- | --- | --- | --- | --- | --- |
|  | **Adult** | | | | | | | | **Pediatric Predicted** | | | | | | | | | |  |
| **Substrate or inhibitor** | **Observed** | | **Predicted** | | **1 to <3 years 15 mg/kg** | | **1 to <3 years 10 mg/kg** | | | | **3 to <6**  **years** | | **6 to <12 years** | | **12 to <18 years** | |  |  |  |
| **Isavuconazole victim DDI** | | | | | | | | | | | | | | | | | | |  |
| **SD ketoconazole** | 1.17 | | 1.17 | | 1.21 | | 1.21 | | | | 1.22 | | 1.20 | | 1.17 | |  |  |  |
|  | (1.10, 1.25) | | (1.16, 1.18) | | (1.19, 1.23) | | (1.19, 1.23) | | | | (1.20, 1.24) | | (1.18, 1.22) | | (1.16, 1.19) | |  |  |  |
| **MD ketoconazole** | 5.22 | | 4.25 | | 2.61 | | 2.61 | | | | 2.79 | | 2.94 | | 3.01 | |  |  |  |
|  | (4.09, 6.66) | | (4.01, 4.50) | | (2.44, 2.80) | | (2.44, 2.80) | | | | (2.60, 2.99) | | (2.74, 3.16) | | (2.81, 3.22) | |  |  |  |
| **MD ketoconazole and isavuconazole** | 1.61 | | 1.62 | | 1.68 | | 1.69 | | | | 1.74 | | 1.78 | | 1.79 | |  |  |  |
|  | (1.50, 1.74) | | (1.59, 1.66) | | (1.62, 1.74) | | (1.63, 1.75) | | | | (1.67, 1.81) | | (1.71, 1.85) | | (1.71, 1.86) | |  |  |  |
| **MD rifampicin and isavuconazole** | 0.102 | | 0.216 | | 0.326 | | 0.315 | | | | 0.305 | | 0.282 | | 0.268 | |  |  |  |
|  | (0.0936, 0.110) | | (0.208, 0.224) | | (0.312, 0.342) | | (0.301, 0.329) | | | | (0.292, 0.319) | | (0.269, 0.296) | | (0.255, 0.281) | |  |  |  |
| **Isavuconazole perpetrator DDI** | | | | | | | | | | | | | | | | | | |  |
| **warfarin** | 1.13 | | 1.11 | | 1.51 | | 1.36 | | | | 1.34 | | 1.37 | | 1.30 | |  |  |  |
|  | (1.03, 1.24) | | (1.10, 1.12) | | (1.48, 1.54) | | (1.34, 1.38) | | | | (1.32, 1.36) | | (1.35, 1.39) | | (1.28, 1.33) | |  |  |  |
| **cyclosporine** | 1.29 | | 1.25 | | 1.54 | | 1.39 | | | | 1.39 | | 1.39 | | 1.31 | |  |  |  |
|  | (1.15, 1.44) | | (1.24, 1.26) | | (1.52, 1.57) | | (1.38, 1.41) | | | | (1.37, 1.40) | | (1.37, 1.40) | | (1.29, 1.33) | |  |  |  |
| **midazolam** | 2.03 | | 1.23 | | 1.52 | | 1.37 | | | | 1.36 | | 1.36 | | 1.31 | |  |  |  |
|  | (1.73, 2.38) | | (1.22, 1.24) | | (1.50, 1.54) | | (1.36, 1.39) | | | | (1.35, 1.38) | | (1.35, 1.38) | | (1.29, 1.32) | |  |  |  |
| **digoxin** | 1.25 | | 1.20 | | 1.38 | | 1.32 | | | | 1.30 | | 1.29 | | 1.24 | |  |  |  |
|  | (1.17, 1.34) | | (1.19, 1.21) | | (1.36, 1.41) | | (1.30, 1.34) | | | | (1.28, 1.31) | | (1.27, 1.31) | | (1.22, 1.26) | |  |  |  |
| **dextromethorphan** | 1.18 | | 1.17 | | na | | na | | | | 1.25 | | 1.25 | | 1.20 | |  |  |  |
|  | (1.02, 1.35) | | (1.15, 1.19) | | na | | na | | | | (1.19, 1.31) | | (1.18, 1.31) | | (1.14, 1.25) | |  |  |  |
| **ethinyl estradiol** | 1.08 | | 1.05 | | na | | na | | | | na | | na | | 1.08 | |  |  |  |
|  | (1.03, 1.13) | | (1.05, 1.05) | | na | | na | | | | na | | na | | (1.07, 1.09) | |  |  |  |
| **rivaroxaban** | 1.13 | | 1.05 | | 1.12 | | 1.09 | | | | 1.09 | | 1.08 | | 1.05 | |  |  |  |
|  | NR | | (1.05, 1.06) | | (1.12, 1.13) | | (1.08, 1.09) | | | | (1.08, 1.09) | | (1.07, 1.08) | | (1.05, 1.06) | |  |  |  |
| **Cmax Ratio** | | | | | | | | | | | | | | | | | | | |
|  | | **Adult** | | | | | | | | **Pediatric Predicted** | | | | | | | | | |
| **Substrate or inhibitor** | | **Observed** | | **Predicted** | | **1 to <3 years 15 mg/kg** | | **1 to <3 years 10 mg/kg** | | | | **3 to <6 years** | | **6 to <12 years** | | **12 to <18 years** | |  |  |
| **Isavuconazole victim DDI** | | | | | | | | | | | | | | | | | | | |
| **SD ketoconazole** | | 1.10 | | 1.04 | | 1.03 | | 1.03 | | | | 1.03 | | 1.03 | | 1.03 | |  |  |
|  | | (0.993, 1.22) | | (1.04, 1.05) | | (1.03, 1.04) | | (1.03, 1.04) | | | | (1.03, 1.03) | | (1.02, 1.03) | | (1.02, 1.03) | |  |  |
| **MD ketoconazole** | | 1.09 | | 1.04 | | 1.03 | | 1.03 | | | | 1.03 | | 1.03 | | 1.03 | |  |  |
|  | | (0.933, 1.27) | | (1.04, 1.05) | | (1.03, 1.04) | | (1.03, 1.04) | | | | (1.03, 1.03) | | (1.03, 1.03) | | (1.02, 1.03) | |  |  |
| **MD ketoconazole and isavuconazole** | | 1.51 | | 1.38 | | 1.33 | | 1.34 | | | | 1.36 | | 1.40 | | 1.41 | |  |  |
|  |  | (1.39, 1.64) | | (1.36, 1.40) | | (1.31, 1.36) | | (1.31, 1.37) | | | | (1.33, 1.39) | | (1.37, 1.43) | | (1.38, 1.45) | |  |  |
| **MD rifampicin and isavuconazole** | | 0.250 | | 0.485 | | 0.673 | | 0.661 | | | | 0.656 | | 0.625 | | 0.598 | |  |  |
|  |  | (0.230, 0.271) | | (0.477, 0.494) | | (0.661, 0.685) | | (0.649, 0.673) | | | | (0.644, 0.669) | | (0.613, 0.637) | | (0.586, 0.611) | |  |  |
| **Isavuconazole perpetrator DDI** | | | | | | | | | | | | | | | | | | | |
| **warfarin** | | 1.05 | | 1.01 | | 1.05 | | 1.04 | | | | 1.04 | | 1.03 | | 1.02 | |  |  |
|  | | (0.911, 1.20) | | (1.01, 1.01) | | (1.04, 1.05) | | (1.03, 1.04) | | | | (1.03, 1.04) | | (1.03, 1.04) | | (1.02, 1.03) | |  |  |
| **cyclosporine** | | 1.06 | | 1.09 | | 1.27 | | 1.20 | | | | 1.21 | | 1.19 | | 1.16 | |  |  |
|  | | (0.953, 1.19) | | (1.09, 1.10) | | (1.26, 1.28) | | (1.20, 1.21) | | | | (1.20, 1.21) | | (1.19, 1.20) | | (1.15, 1.17) | |  |  |
| **midazolam** | | 1.72 | | 1.09 | | 1.31 | | 1.24 | | | | 1.24 | | 1.23 | | 1.19 | |  |  |
|  | | (1.44, 2.05) | | (1.08, 1.10) | | (1.30, 1.33) | | (1.23, 1.24) | | | | (1.23, 1.24) | | (1.22, 1.24) | | (1.18, 1.20) | |  |  |
| **digoxin** | | 1.33 | | 1.14 | | 1.23 | | 1.20 | | | | 1.21 | | 1.22 | | 1.21 | |  |  |
|  | | (1.19, 1.49) | | (1.14, 1.15) | | (1.22, 1.24) | | (1.19, 1.21) | | | | (1.20, 1.22) | | (1.21, 1.23) | | (1.20, 1.22) | |  |  |
| **dextromethorphan** | | 1.17 | | 1.19 | | na | | na | | | | 1.30 | | 1.31 | | 1.26 | |  |  |
|  | | (1.02, 1.35) | | (1.18, 1.21) | | na | | na | | | | (1.27, 1.34) | | (1.27, 1.35) | | (1.23, 1.29) | |  |  |
| **ethinyl estradiol** | | 1.14 | | 1.01 | | na | | na | | | | na | | na | | 1.03 | |  |  |
|  | | (1.03, 1.26) | | (1.01, 1.01) | | na | | na | | | | na | | na | | (1.03, 1.03) | |  |  |
| **rivaroxaban** | | 1.06 | | 1.02 | | 1.04 | | 1.03 | | | | 1.03 | | 1.03 | | 1.02 | |  |  |
|  | | NR | | (1.00, 1.06) | | (1.03, 1.04) | | (1.03, 1.03) | | | | (1.03, 1.03) | | (1.02, 1.03) | | (1.02, 1.02) | |  |  |

Data presented as geometric mean ration (90% confidence interval), na: not applicable, SD: single dose, MD: multiple dose

# Edoxaban

The PBPK model for edoxaban was constructed based on previously reported PBPK models (22, 23). The clinical data utilized in the model building and verification of the edoxaban PBPK model are listed in Table S9. The major metabolite M4 was not included in the present model since M4 is not cleared by P-gp or CYP3A4; therefore, its inclusion was determined to be out of scope for use to verify P-gp inhibition by posaconazole and isavuconazole. The model was first built using single dose iv data from Matsushima, et. al.(24)  and single oral solution mass balance data from Bathala, et. al.(25). The model performance was verified with additional 60 mg single dose oral data and observed DDI data with CYP3A4 and P-gp inhibitors ketoconazole, cyclosporine, verapamil, and quinidine. The final parameters used in the adult edoxaban PBPK model can be found in Table S10. A summary of observed and simulated PK parameters is listed in Table S11. A summary of model performance in DDI studies can be found in Table S12. Visual model performance for 60 mg of edoxaban can be found in **Fig. S4.**

The model was built using ADAM absorption model with Mech P_eff_ model method 1 prediction, full PBPK for distribution, permeability limited liver model for P-gp transport with EHRC. V_ss_ was predicted using Method 2 (Rodgers et al.(26)) and a K_p_ scalar of 1.75 to capture literature reported V_ss_ (107 L) in healthy volunteers receiving 30 mg iv edoxaban (24). Model predicted V_ss_ for 30 mg iv edoxaban was 110 L.

CL_int_ through CYP3A4 was calculated using the built-in Simcyp retrograde calculator, assuming 10% metabolism through CYP3A4(23, 27), CL_iv_ of 21.8 L/h, and renal clearance (CL_R_) of 10.7 L/h (24). The value of CL_int,CYP3A4_ was verified with Simcyp mass balance analysis and reported clearance of metabolites M6 (2.11%) and M8 (0.34%) from the mass balance study(25). Secondarily, CYP3A4 contribution was verified with several clinical DDI studies. Clearance through esterase CES1 was estimated based on the recovery of metabolite M4 (3.5%) from the mass balance study(25). Kinetic clearance through P-gp was included based on published literature and the % available for reabsorption was estimated based on mass balance recovery of unchanged edoxaban in the feces(22, 23, 25). Additional systemic clearance was included to capture clearance of edoxaban through methods not included in the PBPK model (hydrolysis and other unidentified pathways). The PBPK model overestimated the percentage of unchanged edoxaban in the urine (1.42-fold higher) when utilizing the published value of 10.7 L/h. However, it was determined not to impact the predicted DDI with posaconazole and isavuconazole; therefore, it was left unmodified from the published value. A summary of observed and predicted mass balance data can be found in Table S13.

Based on the performance of the edoxaban PBPK model in capturing the observed clinical PK data, observed DDI studies, and mass balance data, it was determined that edoxaban was well verified to be utilized to predict P-gp inhibition by posaconazole and isavuconazole.

Table S9.	Clinical data utilized in the edoxaban compound file development

| **Study (reference)** | **Dose** | **Cohort** | **N** | **Age**  **(years)** | **BMI**  **(kg/m^2^)** |
| --- | --- | --- | --- | --- | --- |
| Single dose (24) | 30 mg iv over 30 min | Healthy  (0.08 female) | 35 | 18-45 | 21-32 |
| Single dose  (24, 27) | 60 mg oral | Healthy  (0.08 female) | 35 | 18-45 | 21-32 |
| Mass balance (25) | 60 mg oral | Healthy male | 6 | 30-55 | 19-29 |
| DDI ketoconazole (28) | day 1-7: 400 mg oral ketoconazole daily  day 4: 60 mg oral edoxaban | Healthy  (0.125 female) | 39 | 18-45 | 18-32 |
| DDI cyclosporine (28) | Single oral dose 500 mg cyclosporine + 60 mg edoxaban | Healthy  (0.125 female) | 39 | 18-45 | 18-32 |
| DDI verapamil (29) | day 1-11: 240 mg SR verapamil  day 10: 60 mg oral edoxaban | Healthy  (0.176 female) | 30 | 31.4 (8.3) | 79.0 (12.8)† |
| DDI quinidine (29) | day 1: 300 mg quinidine  day 2-3: 300 mg oral quinidine Q8H  day 4: 300 mg quinidine  day 3: 60 mg oral edoxaban | Healthy  (16.7 female) | 29 | 30.9 (7.5) | 79.7 (12.0)† |
| DDI quinidine (24) | day 1-3: 300 mg oral quinidine Q8H  day 1: single 30 mg iv edoxaban (over 30 min) | Healthy  (0.08 female) | 28 | 18-45 | 21-32 |

*BSA (m^2^) reported

†weight in kg, mean(SD)

iv: intravenous, min: minute, EM: extensive metabolizer, PM: poor metabolizer, NR: not reported.

Table S10	Input Parameters of the Edoxaban PBPK Model

| Parameter | Value | Assumption(s) and references |
| --- | --- | --- |
| Physicochemical properties and blood binding | | |
| Compound type | Small molecule |  |
| Molecular weight | 548.6 | (22) |
| Log P | 1.61 | (22) |
| Charge | Monoprotic base | (22) |
| pKa 1 | 6.7 | (22) |
| B/P ratio | 0.96 | (22) |
| f_u_ | 0.45 | (22) |
| Plasma binding protein | HSA | (22) |
| **Absorption** | | |
| Absorption Model | ADAM |  |
| f_uGut_ | 0.236 | Predicted by Simcyp |
| P_eff,man_ (10^-4^ cm/s) | 4.61 | Predicted by Simcyp using MechP_eff_ model method 1. P_ttrans,O_ = 112.4 10^-6^ cm/s |
| pH-Solubility (mg/mL) |  | (22, 23) |
| pH 3 | 4.4 |  |
| pH 4 | 4.2 |  |
| pH 5 | 1.8 |  |
| pH 6 | 0.54 |  |
| pH 7 | 0.14 |  |
| Formulation | Immediate Release | Diffusion layer model |
| Transit Time | Segregated transit time |  |
| Distribution | | |
| Distribution model | Full PBPK |  |
| Prediction method of V_ss_ | Method 2 |  |
| V_ss_ (L/kg) | 1.42 | Simcyp predicted |
| K_p_ scalar | 1.75 | Optimized to match observed V_ss_ (24) |
| Elimination | | |
| CYP3A4 CL_int_ (µL/min/pmol isoform) | 0.0034 | Calculated with retrograde calculator  (CL_iv_ 21.8 L/h, f_CL,Bile_ 20%, f_m,CYP3A4_ 10%)(27) |
| CES1 (µL/min/mg protein) | 0.29 | Optimized to match M4 recovery (3.5%) from mass balance study (25) |
| Percentage available for re-absorption (%) | 75 | (23) |
| CL_R_ (L/h) | 10.7 | (24) |
| Additional systemic clearance (L/h) | 1.4 | non-CYP3A4 and CES1 clearance (25) |
| Transporter | | |
| P-gp |  |  |
| J_max_ (pmol/min) | 108 | (22) |
| K_m_ (µM) | 9.1 |  |
| RAF/REF | 0.99 | Set to match default value for Caco-2 cells |
| CL_PD_ - liver (mL/min/million hepatocytes) | 1.01 | Calculated by Simcyp using Mech P_eff_ |

LogP: octanol:buffer partition, B/P ratio: blood-to-plasma partition, f_u_: fraction unbound, HSA: Human Serum Albumin, ADAM: advanced dissolution, absorption and metabolism, f_uGut_: fraction unbound in the gut, P_eff,man_: Human jejunum effective permeability, Mech P_eff_: mechanistic passive reginal permeability, V_ss_: volume of distribution at stead-state, K_p_ scalar: value applied to all tissue:plasma partition coefficients, CL_R_: renal clearance, CL_int_: intrinsic clearance, CL_iv_: intravenous clearance, f_m,CYP3A4_: fraction metabolized CYP3A4, J_max_: in vitro maximum rate of transporter-mediated efflux, K_m_: Michaelis constant, RAF/REF: Relative activity factor or relative expression factor, CL_PD_: passive diffusion clearance

Table S11	Predicted and observed PK parameters for edoxaban in adults

|  | **AUC_inf_ (ng·h/mL)** | | **C_max_ (ng/mL)** | | **CL/F (L/h)** | |
| --- | --- | --- | --- | --- | --- | --- |
|  | **Predicted** | **Observed** | **Predicted** | **Observed** | **Predicted** | **Observed** |
| **30 mg IV** | 1,200  (26.2) | 1,320  (14.4) | 518  (15.4) | 424  (27.1) | 26.9  (28.7) | 21.8  (13.9)* |
| **60 mg PO** | 1,780  (35.5) | 1,770  (24.6) | 304  (38.9) | 256  (34.3) | 39.5  (52.0) | 35.7  (21.4) |
| **60 mg PO (MB study)** | 2,090  (30.8) | 1,600  (19.4) | 367  (35.9) | 332  (32.5) | 32.0  (37.0) | 37.6  (NR) |
| **GMFE** | **1.14** | | **1.17** | | **1.17** | |

*CL

NR: not reported, GMFE: geometric mean fold error, AUC_inf_: area under the curve from 0 to infinity, C_max_: maximum concentration, CL: total clearance (dose/AUC)

data presented as arithmetic mean (%coefficient of variance)

Fig. S4	Predicted and observed plasma concentration curve of oral edoxaban in adult healthy volunteers

Observed data extracted from Parasrampuria, et. al.(27)

Black circles: observed clinical data, solid black line: simulated arithmetic mean, dark grey line: simulated 95th percentile, light grey line: simulated 5th percentile

Table S12	Summary of predicted by the PBPK model and observed PK parameters (AUC and C_max_ ratio) of DDI for edoxaban as the victim

|  | **Without Inhibitor** | | **With Inhibitor** | |  | |
| --- | --- | --- | --- | --- | --- | --- |
|  | **AUC_inf_ (ng*h/mL)** | **C_max_ (ng/mL)** | **AUC_inf_ (ng*h/mL)** | **C_max_ (ng/mL)** | **AUC_inf_ ratio** | **C_max_ Ratio** |
| **400 mg ketoconazole**(28) | | | | | | |
| **Observed** | 1,650 | 245 | 3,000 | 441 | **1.87** | **1.89** |
| **90% CI** |  |  |  |  | (1.76, 1.98) | (1.68, 2.12) |
| **Predicted** | 1,640 | 256 | 3,480 | 504 | **2.12** | **1.97** |
| **90% CI** |  |  |  |  | (2.07, 2.18) | (1.97, 2.01) |
| **Criteria** |  |  |  |  | **1.17 - 2.99** | **1.18 - 3.03** |
| **500 mg cyclosporine** (28) | | | | | | |
| **Observed** | 1,830 | 246 | 3,130 | 411 | **1.73** | **1.74** |
| **90% CI** |  |  |  |  | (1.63, 1.83) | (1.56, 1.93) |
| **Predicted** | 1,640 | 257 | 2,350 | 358 | **1.44** | **1.39** |
| **90% CI** |  |  |  |  | (1.41, 1.46) | (1.36, 1.42) |
| **Criteria** |  |  |  |  | **1.10 - 2.71** | **1.11 - 2.73** |
| **240 mg verapamil SR**(29) ***** | | | | | | |
| **Observed** | 1,450 | 223 | 2,490 | 390 | **1.53** | **1.53** |
| **90% CI** |  |  |  |  | (1.41, 1.65) | 1.34, 1.76) |
| **Predicted** | 1,380 | 259 | 1,940 | 330 | **1.41** | **1.27** |
| **90% CI** |  |  |  |  | (1.38, 1.43) | (1.26, 1.29) |
| **Criteria** |  |  |  |  | **1.01 - 2.3** | **1.01 - 2.31** |
| **300 mg quinidine (oral edoxaban)**(29) ***** | | | | | | |
| **Observed** | 1,440 | 226 | 2,110 | 320 | **1.77** | **1.85** |
| **90% CI** |  |  |  |  | (1.65, 1.89) | (1.65, 2.09) |
| **Predicted** | 1,590 | 289 | 2,520 | 494 | **1.59** | **1.71** |
| **90% CI** |  |  |  |  | (1.56, 1.61) | (1.67, 1.74) |
| **Criteria** |  |  |  |  | **1.12 - 2.79** | **1.16 - 2.95** |
| **300 mg quinidine (iv edoxaban)**(24) | | | | | | |
| **Observed** | 1,390 | 438 | 1,870 | 471 | **1.35** | **1.07** |
| **90% CI** |  |  |  |  | (1.28, 1.43) | (0.963, 1.20) |
| **Predicted** | 1,120 | 509 | 1,460 | 580 | **1.30** | **1.14** |
| **90% CI** |  |  |  |  | (1.29, 1.32) | (1.13, 1.14) |
| **Criteria** |  |  |  |  | **0.935 - 1.95** | **0.824 - 1.39** |

*AUC_0-24h_: area under the curve from 0 to 24 hour

AUC_inf_: area under the curve from 0 to infinity, C_max_: maximum concentration, CI: confidence interval, iv: intravenous

AUC ratio: AUC(with inhibitor)/AUC(without inhibitor), C_max_ ratio: C_max_(with inhibitor)/C_max_(without inhibitor)

Criteria determined by GUEST method assuming 20% variability in pharmacokinetic parameters(21)

Table S13	Edoxaban mass balance analysis from clinical study and predicted by the PBPK model

|  | **% Feces** | | **%Urine** | | **%Total Recovery** | |
| --- | --- | --- | --- | --- | --- | --- |
| **Analyte** | **Predicted** | **Observed** | **Predicted** | **Observed** | **Predicted** | **Observed** |
| % of dose | - | 62.2 | - | 35.4 | 100 | 97.6 |
| Edoxaban | 53.1 | 49.1 | 33.9 | 23.8 | 87.0 | 72.8 |
| CYP3A4 metabolism | - | 2.00 | - | 0.45 | 2.80 | 2.45 |
| CES1 metabolism | - | 1.93 | - | 1.56 | 5.32 | 3.49 |
| Additional CL | - | 0.05 | - | 3.22 | 4.58 | 3.27 |

# References

1. FDA. CRESEMBA prescribing label.

2. Cresemba (isavuconazonium sulfate). <https://www.cresemba.com/>. Retrieved 28 October 2025.

3. Liu Y, Li J, Zhu H-J. 2024. Regulation of carboxylesterases and its impact on pharmacokinetics and pharmacodynamics: an up-to-date review. Expert Opin Drug Metab Toxicol 20:377–397.

4. Pope CN, Karanth S, Liu J, Yan B. 2005. Comparative carboxylesterase activities in infant and adult liver and their in vitro sensitivity to chlorpyrifos oxon. Regul Toxicol Pharmacol 42:64–69.

5. Desai A, Kovanda L, Kowalski D, Lu Q, Townsend R, Bonate PL. 2016. Population Pharmacokinetics of Isavuconazole from Phase 1 and Phase 3 (SECURE) Trials in Adults and Target Attainment in Patients with Invasive Infections Due to Aspergillus and Other Filamentous Fungi. Antimicrob Agents Chemother 60:5483–5491.

6. Townsend R, Kato K, Hale C, Kowalski D, Lademacher C, Yamazaki T, Akhtar S, Desai A. 2018. Two Phase 1, Open‐Label, Mass Balance Studies to Determine the Pharmacokinetics of 14C‐Labeled Isavuconazonium Sulfate in Healthy Male Volunteers. Clin Pharmacol Drug Dev 7:207–216.

7. Townsend RW, Akhtar S, Alcorn H, Berg JK, Kowalski DL, Mujais S, Desai AV. 2017. Phase I trial to investigate the effect of renal impairment on isavuconazole pharmacokinetics. Eur J Clin Pharmacol 73:669–678.

8. Ford SL, Sutton K, Lou Y, Zhang Z, Tenorio A, Trezza C, Patel P, Spreen W. 2017. Effect of Rifampin on the Single-Dose Pharmacokinetics of Oral Cabotegravir in Healthy Subjects. Antimicrob Agents Chemother 61:10.1128/aac.00487-17.

9. Townsend R, Dietz A, Hale C, Akhtar S, Kowalski D, Lademacher C, Lasseter K, Pearlman H, Rammelsberg D, Schmitt‐Hoffmann A, Yamazaki T, Desai A. 2017. Pharmacokinetic Evaluation of CYP3A4‐Mediated Drug‐Drug Interactions of Isavuconazole With Rifampin, Ketoconazole, Midazolam, and Ethinyl Estradiol/Norethindrone in Healthy Adults. Clin Pharmacol Drug Dev 6:44–53.

10. Wong M, Balleine RL, Collins M, Liddle C, Clarke CL, Gurney H. 2004. CYP3A5 Genotype and Midazolam Clearance in Australian Patients Receiving Chemotherapy. Clin Pharmacol Ther 75:529–538.

11. Heizmann P, Eckert M, Ziegler W. 1983. Pharmacokinetics and bioavailability of midazolam in man. Br J Clin Pharmacol 16:43S-49S.

12. Paine MF, Shen DD, Kunze KL, Perkins JD, Marsh CL, McVicar JP, Barr DM, Gillies BS, Thummel KE. 1996. First-pass metabolism of midazolam by the human intestine. Clin Pharmacol Ther 60:14–24.

13. Schmitt-Hoffmann A, Roos B, Heep M, Schleimer M, Weidekamm E, Brown T, Roehrle M, Beglinger C. 2006. Single-Ascending-Dose Pharmacokinetics and Safety of the Novel Broad-Spectrum Antifungal Triazole BAL4815 after Intravenous Infusions (50, 100, and 200 Milligrams) and Oral Administrations (100, 200, and 400 Milligrams) of Its Prodrug, BAL8557, in Healthy Volunteers. Antimicrob Agents Chemother 50:279–285.

14. Schmitt-Hoffmann A, Roos B, Maares J, Heep M, Spickerman J, Weidekamm E, Brown T, Roehrle M. 2006. Multiple-Dose Pharmacokinetics and Safety of the New Antifungal Triazole BAL4815 after Intravenous Infusion and Oral Administration of Its Prodrug, BAL8557, in Healthy Volunteers. Antimicrob Agents Chemother 50:286–293.

15. Segers H, Deville JG, Muller WJ, Manzanares A, Desai A, Neely M, Bordon V, Hanisch B, Lassaletta A, Fisher BT, Autmizguine J, Groll AH, Sinnar S, Croos-Dabrera R, Engelhardt M, Jones M, Kovanda LL, Arrieta AC. 2024. Safety, outcomes, and pharmacokinetics of isavuconazole as a treatment for invasive fungal diseases in pediatric patients: a non-comparative phase 2 trial. Antimicrob Agents Chemother 68:e00484-24.

16. Desai A, Yamazaki T, Dietz AJ, Kowalski D, Lademacher C, Pearlman H, Akhtar S, Townsend R. 2017. Pharmacokinetic and Pharmacodynamic Evaluation of the Drug‐Drug Interaction Between Isavuconazole and Warfarin in Healthy Subjects. Clin Pharmacol Drug Dev 6:86–92.

17. Groll AH, Desai A, Han D, Howieson C, Kato K, Akhtar S, Kowalski D, Lademacher C, Lewis W, Pearlman H, Mandarino D, Yamazaki T, Townsend R. 2017. Pharmacokinetic Assessment of Drug‐Drug Interactions of Isavuconazole With the Immunosuppressants Cyclosporine, Mycophenolic Acid, Prednisolone, Sirolimus, and Tacrolimus in Healthy Adults. Clin Pharmacol Drug Dev 6:76–85.

18. Yamazaki T, Desai A, Goldwater R, Han D, Lasseter KC, Howieson C, Akhtar S, Kowalski D, Lademacher C, Rammelsberg D, Townsend R. 2017. Pharmacokinetic Interactions Between Isavuconazole and the Drug Transporter Substrates Atorvastatin, Digoxin, Metformin, and Methotrexate in Healthy Subjects. Clin Pharmacol Drug Dev 6:66–75.

19. Yamazaki T, Desai A, Goldwater R, Han D, Howieson C, Akhtar S, Kowalski D, Lademacher C, Pearlman H, Rammelsberg D, Townsend R. 2017. Pharmacokinetic Effects of Isavuconazole Coadministration With the Cytochrome P450 Enzyme Substrates Bupropion, Repaglinide, Caffeine, Dextromethorphan, and Methadone in Healthy Subjects. Clin Pharmacol Drug Dev 6:54–65.

20. Rohr BS, Foerster KI, Blank A, Burhenne J, Mahmoudi M, Haefeli WE, Mikus G. 2022. Perpetrator Characteristics of Azole Antifungal Drugs on Three Oral Factor Xa Inhibitors Administered as a Microdosed Cocktail. Clin Pharmacokinet 61:97–109.

21. Guest EJ, Aarons L, Houston JB, Rostami-Hodjegan A, Galetin A. 2011. Critique of the Two-Fold Measure of Prediction Success for Ratios: Application for the Assessment of Drug-Drug Interactions. Drug Metab Dispos 39:170–173.

22. Xu R, Liu W, Ge W, He H, Jiang Q. 2023. Physiologically‐based pharmacokinetic pharmacodynamic parent‐metabolite model of edoxaban to predict drug–drug‐disease interactions: M4 contribution. CPT: Pharmacomet Syst Pharmacol 12:1093–1106.

23. Kato T, Mikkaichi T, Yoshigae Y, Okudaira N, Shimizu T, Izumi T, Ando S, Matsumoto Y. 2021. Quantitative analysis of an impact of P-glycoprotein on edoxaban’s disposition using a human physiologically based pharmacokinetic (PBPK) model. Int J Pharm 597:120349.

24. Matsushima N, Lee F, Sato T, Weiss D, Mendell J. 2013. Bioavailability and Safety of the Factor Xa Inhibitor Edoxaban and the Effects of Quinidine in Healthy Subjects. Clin Pharmacol Drug Dev 2:358–366.

25. Bathala MS, Masumoto H, Oguma T, He L, Lowrie C, Mendell J. 2012. Pharmacokinetics, Biotransformation, and Mass Balance of Edoxaban, a Selective, Direct Factor Xa Inhibitor, in Humans. Drug Metab Dispos 40:2250–2255.

26. Rodgers T, Rowland M. 2007. Mechanistic Approaches to Volume of Distribution Predictions: Understanding the Processes. Pharm Res 24:918–933.

27. Parasrampuria DA, Truitt KE. 2016. Pharmacokinetics and Pharmacodynamics of Edoxaban, a Non-Vitamin K Antagonist Oral Anticoagulant that Inhibits Clotting Factor Xa. Clin Pharmacokinet 55:641–655.

28. Parasrampuria DA, Mendell J, Shi M, Matsushima N, Zahir H, Truitt K. 2016. Edoxaban drug–drug interactions with ketoconazole, erythromycin, and cyclosporine. Br J Clin Pharmacol 82:1591–1600.

29. Mendell J, Zahir H, Matsushima N, Noveck R, Lee F, Chen S, Zhang G, Shi M. 2013. Drug-Drug Interaction Studies of Cardiovascular Drugs Involving P-Glycoprotein, an Efflux Transporter, on the Pharmacokinetics of Edoxaban, an Oral Factor Xa Inhibitor. Am J Cardiovasc Drugs 13:331–342.
